# Hericenone C exhibits anti-nociceptive effects through RORα-mediated suppression of TLR4 transcription

**DOI:** 10.3389/fphar.2026.1703176

**Published:** 2026-03-18

**Authors:** Junhao Li, Kengo Hamamura, Yuya Yoshida, Shimpei Kawano, Shohei Uchinomiya, Jiahongyi Xie, Damiana Scuteri, Yang Ruan, Tomohito Tanihara, Kohei Fukuoka, Orion Zaitsu, Fumiaki Tsurusaki, Ryotaro Tsukamoto, Takumi Nishi, Taiki Fukuda, Tsukasa Hamasaki, Kosuke Oyama, Giacinto Bagetta, Akio Ojida, Kuniyoshi Shimizu, Chaofeng Zhang, Shigehiro Ohdo, Naoya Matsunaga

**Affiliations:** 1 Department of Clinical Pharmacokinetics, Faculty of Pharmaceutical Sciences, Kyushu University, Fukuoka, Japan; 2 Department of Medical Chemistry and Chemical Biology, Faculty of Pharmaceutical Sciences, Kyushu University, Fukuoka, Japan; 3 Department of Agro-Environmental Sciences, Graduate School of Bioresources and Bioenvironmental Sciences, Kyushu University, Fukuoka, Japan; 4 Department of Health Sciences, Magna Graecia University of Catanzaro, Catanzaro, Italy; 5 Joint International Research Laboratory of Target Discovery and New Drug Innovation, Ministry of Education, School of Traditional Chinese Medicines, China Pharmaceutical University, Nanjing, China; 6 Sino-Jan Joint Lab of Natural Health Products Research, School of Traditional Chinese Medicines, China Pharmaceutical University, Nanjing, China; 7 Department of Biological Science and Technology, Tokyo University of Science, Tokyo, Japan; 8 Pharmacotechnology Documentation and Transfer Unit, Preclinical and Translational Pharmacology, Department of Pharmacy, Health and Nutritional Sciences, University of Calabria, Rende, Italy

**Keywords:** CD11c, formalin test, hericenone C, NF-κB, nociceptive behavior, p65, RORα, TLR4

## Abstract

**Introduction:**

Hericenone C exhibits antinociceptive effects in inflammatory pain; however, its molecular target and underlying mechanism remain unclear.

**Methods and Results:**

We assessed the effect of hericenone C on formalin-induced nociceptive behavior in mice and explored its molecular target using *in vitro* experiments. Based on competitive affinity proteomics, we identified direct interactions between RORα and hericenone C; functional assays confirmed the role of hericenone C as a RORα antagonist that suppresses RORE-mediated transcriptional activity. Integrated bioinformatics and experimental validation indicated hericenone C-mediated suppression of TLR4 expression *via* inhibited RORα binding to the TLR4 promoter, which attenuates NF-κB signaling. This mechanism was further validated through pharmacological and genetic approaches, revealing that hericenone C and RORα antagonist SR3335 synergistically modulate TLR4 expression in RORα-modified macrophages. In the formalin-induced nociceptive pain model mice, formalin activated NF-κB through TLR4-dependent P65 phosphorylation, while macrophage depletion selectively suppressed phase 2 nociception. Critically, adoptive transfer of RORα-overexpressing or SR1078-pretreated monocyte-enriched PBMCs exacerbated pain, which was effectively reversed by hericenone C. Notably, hericenone C pretreatment reduced CD11c^+^ cell infiltration and decreased TLR4 expression in inflamed paw tissues.

**Conclusion:**

Overall, these findings establish hericenone C as a novel RORα antagonist that alleviates inflammatory pain through inhibition of the RORα-TLR4-NF-κB axis in CD11c^+^ cells, offering a promising therapeutic strategy for pain management.

## Graphical Abstract

**Figure:**
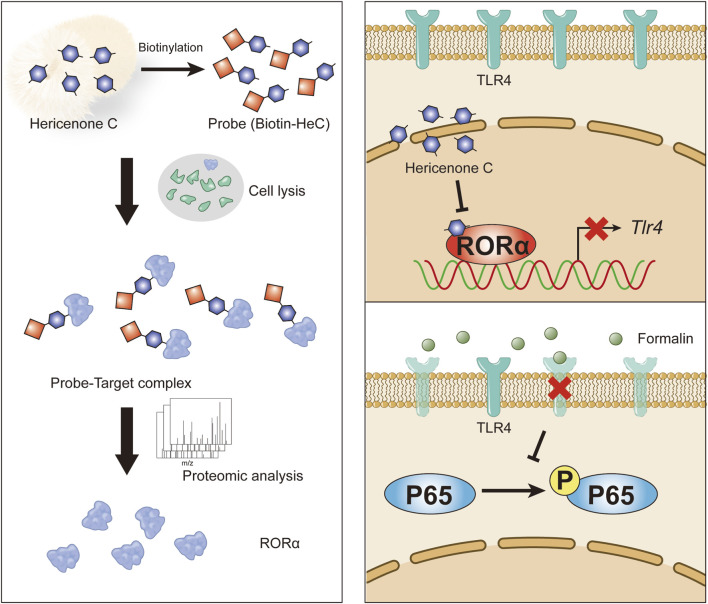

## 1 Introduction

Natural products represent a rich source of bioactive compounds with pharmaceutical potential. *Hericium erinaceus* (lion’s mane mushroom), a medicinal fungus in traditional Chinese medicine, has been well explored due to its diverse therapeutic properties, including neuroprotective, anti-inflammatory, and immunomodulatory effects (Friedman, 2015; Szućko-Kociuba et al., 2023). Chemical analyses of *H. erinaceus* reveal a wide array of bioactive constituents, such as polysaccharides, erinacines, and hericenones, which contribute to its pharmacological activities. Notably, hericenones, a class of orsellinic acid-derived meroterpenoids, exhibit unique structural features and biological functions (Han et al., 2025; Kawagishi, 2018). Hericenone C exhibits neurotrophic effects *via* enhanced nerve growth factor (NGF) and brain-derived neurotrophic factor (BDNF) expression (Kawagishi et al., 1991; Kobayashi et al., 2021; Tamrakar et al., 2023). Although the antinociceptive and anti-inflammatory effects of hericenone C are evident (Li et al., 2024), its binding target remains unclear.

The formalin test involves a well-established and widely used model for studying chemical stimulation-mediated persistent pain, particularly in evaluating analgesic mechanisms and drug efficacy (Xinqiang et al., 2022). This model elicits a biphasic nociceptive response: Phase 1 (0–15 min post-injection) represents acute neurogenic pain, primarily driven by direct chemical activation of C-fiber nociceptors and TRPA1 channels, reflecting an immediate but brief pain response (Sepulveda et al., 2022). Phase 2 (15–60 min post-injection) involves tonic inflammatory pain, characterized by peripheral tissue inflammation and central sensitization, which is mediated by the release of pro-inflammatory cytokines and spinal cord hyperexcitability. Notably, phase 1 is sensitive to centrally acting analgesics, such as opioids, while phase 2 responds to both central and peripheral agents, including non-steroid anti-inflammatory drugs (NSAIDs) (Gwon et al., 2021). The formalin test, exhibiting a biphasic nature, provides a robust platform for investigating both acute and chronic inflammatory pain mechanisms, making it particularly valuable for screening novel analgesics and deciphering their modes of action. Previously, we reported that hericenone C alleviated the second phase of formalin-induced nociceptive behaviors (Li et al., 2024); however, the underlying molecular mechanism remains unclear.

Therefore, in this study, we aimed to identify the direct molecular targets of hericenone C and to elucidate the signaling mechanisms underlying inflammatory pain modulation by hericenone C.

## 2 Materials and methods

### 2.1 Animals

All animal experiments were conducted following the guidelines of Kyushu University, Japan, and were approved by its Institutional Animal Care and Use Committee (approval no. A25-182-0; granted on 5 December 2024). Four-week-old male *ICR* mice acquired from Charles River Laboratories, Inc. were housed in a controlled environment (24 °C ± 2 °C, 60% ± 10% humidity), with access to water and standard pelleted diet *ad libitum*. Only male mice were used, considering previously reported effects of the estrous phase on female behavior in the second phase of the formalin test (Pepino et al., 2023).

### 2.2 Isolation of monocyte-enriched peripheral blood mononuclear cells (PBMCs)

PBMCs were isolated from mouse whole blood using Lymphoprep density gradient medium (StemCell Technologies, Vancouver, Canada) following the instructions provided by the manufacturer. For this experiment, blood was carefully layered onto an equal volume of Lymphoprep and centrifuged at 800 *g* for 20 min at room temperature with the brake off. The PBMC layer at the interface was collected and washed twice with saline. To enrich monocytes, the freshly isolated PBMCs were resuspended in complete RPMI-1640 medium and incubated for 1 h at 37 °C in a 5% CO_2_ incubator to enable adherence to culture plates. Subsequently, non-adherent cells were removed by gentle washing with PBS. The adherent cell population, representing monocyte-enriched PBMCs, was then harvested for subsequent experimental procedures through gentle cell scraping (Zhang et al., 2017). The isolated monocyte-enriched PBMCs were either treated with 20 µM SR1078 for 12 h, or transfected with pcDNA or RORα-expressing plasmids.

### 2.3 TLR4 promoter reporter vector construction

Genomic DNA was extracted from the mouse tail tissue. Different fragments of the mouse Tlr4 gene promoter region (from −1,691, −1,272, −937, −529, or −114 to +154 bp) were amplified *via* PCR using PrimeSTAR MAX DNA Polymerase (Takara Bio Co., Ltd., Osaka, Japan). The purified PCR products were ligated into the pGL4.18 luciferase reporter vector (Promega Corporation, WI, USA) using a DNA Ligation Kit (Nippon Gene). The primers used for amplification are listed in Supplementary Table S1.

### 2.4 Formalin test and hericenone C treatments

Hericenone C (10 mg/kg) was isolated and purified as previously described (Tamrakar et al., 2023), with its structural identity and purity (>99% by HPLC) confirmed (Supplementary Figure S13). For the *in vivo* experiment, hericenone C was suspended in corn oil (Nacalai Tesque, Kyoto, Japan); it was administered intraperitoneally (*i.p.*) 45 min before formalin injection. For assessing the formalin-induced nociceptive behavior, 20 μL of 2.5% formalin (FUJIFILM Wako Pure Chemical Corporation, Osaka, Japan) (diluted in saline) was administered to the right hindfoot (intraplantar: *i.pl.*) of mice (Katsuyama et al., 2015; Scuteri et al., 2018). Next, SR1078-treated or transfected PBMCs were harvested and resuspended in sterile PBS (2 × 10^5^ cells/mL); 10 µL of this cell suspension was subcutaneously injected at a different site on the same hind paw of each mouse 5 min before formalin injection. The duration of licking and biting at the administration site was recorded at 5-min intervals for 45 min after administration. Behavioral assessments were conducted under single-blind conditions.

### 2.5 Cell culture and transient transfection

RAW264.7 mouse macrophage-like cells (RRID: CVCL_0493) and NIH3T3 fibroblasts (RRID: CVCL_0594) were purchased from American Type Culture Collection (VA, USA) and Cell Resource Center for Biomedical Research (Tohoku University, Sendai, Japan), respectively. Cells were cultured in DMEM medium (Life Technologies, Carlsbad, CA) supplemented with 10% heat-inactivated fetal bovine serum (FBS) and 0.5% penicillin-streptomycin solution (Invitrogen; Life Technologies, Carlsbad, California, USA) in a humidified incubator maintaining 5% CO_2_ at 37 °C (Tsuruta et al., 2022; Yoshida et al., 2021). All experiments were conducted with cells that had been used for less than 3 months after resuscitation from authenticated frozen stocks. Cell line identity was confirmed by the suppliers based on short tandem repeat (STR) profiling. Routine *mycoplasma* testing was performed using the MycoBlue *Mycoplasma* Detector (Vazyme Biotech, Nanjing, China) as previously described to confirm the absence of contamination in cultures (Tsuruta et al., 2022).

RAW264.7 cells were cultured to achieve 70%–80% confluence, washed with PBS, and resuspended in antibiotic-free Opti-MEM. Transfections were performed using the Nepa21 electroporation system (Nepa Gene, Chiba, Japan). For RORα overexpression, 1 µg of RORα-expressing plasmid (VectorBuilder Inc., Chicago, IL, USA) or empty pcDNA3.1 vector (Invitrogen) was used per 1 × 10^6^ cells. For gene knockdown, 100 pmol of the different siRNAs (Invitrogen) was used per 1 × 10^6^ cells: a non-targeting control (siControl), a mouse RORα-targeting siRNA (siRORα), and a mouse TLR4-targeting siRNA (siTLR4). The optimized electroporation parameters were as follows: Poring Pulse – 175 V, pulse length 5 ms, pulse interval 50 ms, two pulses; Transfer Pulse – 20 V, pulse length 50 ms, pulse interval 50 ms, two pulses.

Freshly isolated monocyte-enriched PBMCs were transfected using Lipofectamine LTX with PLUS Reagent (Invitrogen) according to the instructions provided by the manufacturer. For transfection in 6-well plates, cells were resuspended in complete RPMI-1640 medium without antibiotic supplementation. In each well, a transfection mixture comprising 1 µg of either RORα-expressing plasmid (VectorBuilder) or empty pcDNA3.1 vector control, complexed with the Lipofectamine LTX/PLUS reagent system.

### 2.6 Luciferase reporter assay

For the luciferase reporter assay, RORα response element (RORE)-driven luciferase reporter plasmid (RORE::Luc) (Promega) and pRL-TK (Promega) were cotransfected into the cells *via* electroporation as above, followed by 40 µM of hericenone C treatment.

NIH3T3 cells were seeded at a density of 2.0 × 10^5^ cells per well in 24-well plates. Cells were transfected using Lipofectamine LTX with PLUS Reagent (Invitrogen) according to the manufacturer’s protocol. Each well received a transfection mixture containing 200 ng of TLR4 promoter-luciferase reporter vector, 500 ng of either RORα expression plasmid (VectorBuilder) or empty pcDNA3.1 vector, and 10 ng of pRL-TK vector (Promega) as an internal control. At 24-h post-transfection, cells were treated with 40 μM hericenone C for 1 h.

Luciferase activity was measured using the Dual-Luciferase^®^ Reporter Assay System (Promega), and firefly luciferase activity was normalized to Renilla luciferase activity for each sample.

### 2.7 Quantitative RT-PCR

Quantitative RT-PCR analysis was performed following previously described methods (Yoshida et al., 2021, 2024). Briefly, total RNA was extracted using RNAiso (Takara), and cDNA was synthesized using a ReverTra Ace qPCR RT kit (Toyobo, Osaka, Japan). Subsequently, RT-PCR analysis was performed on diluted cDNA samples using THUNDERBIRD SYBR qPCR Mix (Toyobo) with a 7500 Real-time PCR system (Applied Biosystems, CA, USA). Data were normalized using 18S rRNA mRNA as controls. Supplementary Table S1 presents the list of primer sequences used in this study.

### 2.8 Western blotting

Western blotting was performed following previously described methods (Yoshida et al., 2021, 2024). Primary antibodies against P65 (ab16502, Abcam, Cambridge, UK) and p-P65 (3033S; Cell Signaling Technology, Inc., MA, USA) were used; an anti-rabbit IgG (ab205718, Abcam) was used as secondary antibody. Immunoreactive bands were visualized using ImmunoStar LD (FUJIFILM Wako Pure Chemical Corporation), and chemiluminescent signals were developed using FUSION FX (M&S Instruments Inc., Osaka, Japan) and were semi-quantitatively assessed based on the P65 levels.

### 2.9 Pull-down assay

The biotinylated hericenone C was synthesized at the Department of Medical Chemistry and Chemical Biology, Faculty of Pharmaceutical Sciences, Kyushu University. The RAW264.7 cells were collected and washed with ice-cold PBS. Next, the subcellular fractionation of RAW264.7 cells was performed using SHE buffer (10 mM HEPES [pH 7.5], 210 mM mannitol, 70 mM sucrose, 1 mM EDTA, 1 mM EGTA) for cytosolic lysis, followed by nuclear lysis with NE buffer (50 mM HEPES [pH 7.5], 0.35 M NaCl, 0.1% NP40). Protein concentration was normalized through the BCA assay. For target enrichment, both lysates were pre-incubated with 100 µM hericenone C for 1 h at 4 °C, followed by overnight incubation with biotinylated hericenone C at 4 °C. Subsequently, MS300 streptavidin beads (5325, Takara) were added to the lysates, and the complexes were incubated overnight at 4 °C. After washing with wash buffer (50 mM HEPES [pH 7.5], 0.15 M NaCl, 0.1%NP-40). The bound proteins were obtained for LC-MS analysis or Western blotting.

### 2.10 Proteomic analysis by LC-MS

For the LC-MS-based proteomic analysis, protein-bound beads were resuspended in 50 µL of 6 M urea/50 mM ammonium bicarbonate buffer. Subsequently, 300 µL of acetone was added, and the mixture was incubated at −30 °C for 2 h. The supernatant was separated from the beads using a magnetic rack and discarded after centrifugation at ×12,000 g and 4 °C for 15 min. The resulting pellet was resuspended in 100 µL of 6 M urea/50 mM ammonium bicarbonate buffer. Subsequently, 2 µL of 500 mM dithiothreitol (DTT) was added, and the sample was incubated at 37 °C for 2 h. After cooling to room temperature, 4 µL of 500 mM iodoacetamide (IAA) was added, and the mixture was incubated in the dark for 45 min. The alkylation reaction was quenched by adding 4 µL of 50 mM DTT, followed by incubation for 30 min. Next, 400 µL of 50 mM ammonium bicarbonate buffer was added, and trypsin (AB Sciex, Parts no. 4352157) was added at a ratio of 1:50 (trypsin:protein, w/w), followed by overnight digestion at 37 °C. The digest was acidified with 10% formic acid to pH ≈ 4. Desalting was performed using a GL-Tip SDB (GL Science) following the instructions provided by the manufacturer, and solvents were removed under low-pressure evaporation. The final sample was dissolved in 0.1% formic acid to a concentration of 1.0 μg/μL for LC-MS analysis.

Peptide samples were separated using an Eksigent NanoLC Ekspert 400 (Sciex) coupled online to a TripleTOF 6600 (Sciex). The mobile phases consisted of solvent A (0.1% formic acid in water) and solvent B (0.1% formic acid in acetonitrile). Peptides were loaded onto a C18 analytical column (Chrom nanoLC column 75 μm id × 15 cm, Sciex) and eluted with a 140-min linear gradient from 2% to 80% solvent B at a flow rate of 300 nL/min. The mass spectrometer was operated in data-dependent acquisition (DDA) mode. Full MS scans (MS1) were acquired over a range of 400–1,250 m/z with an accumulation time of 0.25 s. MS/MS scans (MS2) were performed over a range of 100–1,500 m/z with an accumulation time of 0.025 s, an isolation width of approximately 0.7 Da, and rolling collision energy with a collision energy spread of 5. Dynamic exclusion was set to 30 s to prevent repeated sequencing of the same peptides. Raw data were processed using ProteinPilot software (version 5.0.2, Sciex). Database searches were conducted against the Uniprot *Mus musculus* (Mouse) database (release July 2020), supplemented with a contaminants database. The search parameters were set as follows: enzyme, trypsin; cysteine alkylation, iodoacetamide; variable modifications, biological modifications. Protein identification and quantification were performed using the Paragon algorithm within the software.

### 2.11 Chromatin immunoprecipitation (ChIP)

RAW264.7 cells were fixed by incubating them with 10% formaldehyde for 20 min at room temperature to facilitate chromatin crosslinking; the reaction was quenched by adding glycine (0.25 M). The crosslinked samples were sonicated on ice using a Bioruptor (Diagenode, Liège, Belgium) and immunoprecipitated using anti-RORα antibody or rabbit IgG (Santa Cruz Biotechnology) used as a control. DNA was purified using the QIAquick PCR Purification Kit (Qiagen, Valencia, CA, USA). PCR amplification was performed with GoTaq Green Master Mix (Promega). The products were resolved on a 1.5% agarose gel, stained with ethidium bromide, and visualized using an ImageQuant LAS 3000 mini imaging system (Fuji Film). All data were normalized to the input DNA PCR products.

### 2.12 Fluorescent immunostaining

The fluorescent immunostaining assay was performed following previously described methods (Hamamura et al., 2020). Frozen paw sections (20 μm thick) were sliced using a CryostarNX70 (Thermo Fisher Scientific, MA, USA). Primary antibody reactions using anti-TLR4 (14358S; Cell Signaling Technology, Inc.) and anti-CD11c-APC (BL-117352, BioLegend) were conducted at a 1:1,000 dilution for 3 days at 4 °C. The incubation with secondary antibodies, including goat anti-rabbit IgG H&L (Alexa Fluor^®^ 647) (ab150083: Abcam) for anti-TLR4 (1:1,000), was performed for 3 h at room temperature. Sections were processed using the Vector TrueVIEW^®^ Autofluorescence Quenching Kit (SP-8500: Vector Laboratories, Inc., CA, USA) and mounted (H-2000: Vector Laboratories). Subsequently, imaging was performed using a Keyence BZ-800 fluorescence microscope (Keyence, Osaka, Japan).

### 2.13 Statistical analysis

Data are presented as mean ± standard deviation (SD) deduced from at least three independent experiments, as indicated in the figure legends. Statistical analyses were performed using JMP Pro 17 software (SAS Institute Japan, Tokyo, Japan). The normality of data distribution was evaluated using the Shapiro–Wilk test. Data were compared between the two groups using an unpaired two-tailed Student’s t-test. For comparisons among multiple groups, one-way analysis of variance (ANOVA) followed by Tukey’s *post hoc* test was applied. Statistical significance was considered at *p*-value < 0.05 (*p < 0.05, **p < 0.01).

## 3 Results

### 3.1 RORα is identified as the binding target of hericenone C

**FIGURE 1:**
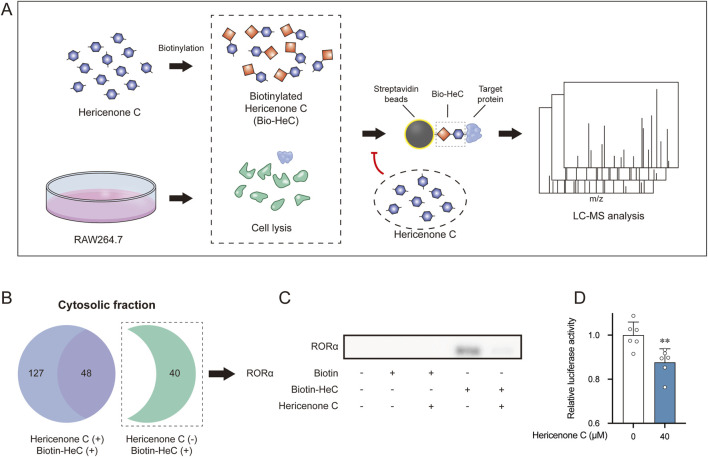
Identification of the binding target of hericenone C. **(A)** Screening the binding target through LC-MS. Hericenone C, a competitive inhibitor, was used for a 1-h pre-treatment to exclude the unspecific binding targets. **(B)** Venn diagram of the binding targets in the cytosolic fraction. **(C)** Direct binding confirmation between hericenone C and RORα by Western blotting. **(D)** Effect of hericenone C on RORE::Luc cells. RORE::Luc cells was treated for 1 h with hericenone **(C)**. Each value represents the mean ± SD derived from six independent experiments (*n* = 6). *p < 0.05 compared the 40 μM hericenone C group with the 0 µM of hericenone C group using unpaired Student’s t-test.

Considering the antinociceptive effects of hericenone C in the second phase of formalin-induced pain (Li et al., 2024), we explored its molecular target responsible underlying this pharmacological activity. The competitive affinity-based proteomic profiling using biotinylated hericenone C, with unlabeled hericenone C serving as a competitive inhibitor, revealed 102 nuclear and 40 cytosolic candidate binding proteins in cytosolic fractions (Figure 1A; Supplementary Figure S1). Among these, we focused on retinoic acid receptor-related orphan receptor alpha (RORα) from the cytosolic fraction for further validation owing to its involvement in inflammatory regulation. Western blot analysis confirmed the direct interaction of hericenone C with RORα (Figure 1C). Furthermore, we characterized the pharmacological activity of hericenone C on RORα through luciferase reporter assays using RAW264.7 cells transfected with an RORE::Luc plasmid. Notably, hericenone C treatment significantly suppressed RORE-mediated luciferase activity (Figure 1D), suggesting that hericenone C acts as a RORα antagonist.

### 3.2 Hericenone C suppresses *Tlr4* transcription *via* RORα

**FIGURE 2:**
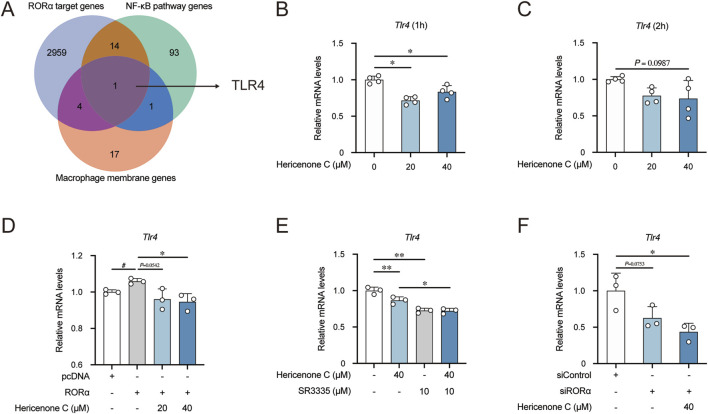
Effect of hericenone C on TLR4 regulation *via* targeting RORα. **(A)** Venn diagram for TLR4 screening as a downstream target of RORα. **(B,C)** Effect of hericenone C on *Tlr4* mRNA expression for **(B)** 1 h and **(C)** 2 h. **(D)** Hericenone C-mediated regulation of *Tlr4* mRNA expression in RORα-overexpressing RAW264.7 cells. **(E)** Effect of hericenone C and SR3335 on *Tlr4* mRNA expression. **(F)** Hericenone C-mediated regulation of *Tlr4* mRNA expression in RORα-knockdown RAW264.7 cells. In all panels, each value represents the mean ± SD from three or four independent experiments (*n* = 3–4). **(B,C)** *p < 0.05 compared to 0 μM of the hericenone C group by Dunnett’s test. **(D)** ^#^ *p* < 0.05 compared the RORα-overexpression group with the pcDNA group by unpaired Student’s t-test. *p < 0.05 compared to the RORα-overexpression group using Dunnett’s test. **(E)** **p < 0.01 compared to 0 μM of the hericenone C group through Dunnett’s test. *p < 0.05 compared the 40 µM of hericenone C and 10 µM SR3335 group with the 40 µM of hericenone C group through Student’s t-test. **(F)** *p < 0.05 compared to the siControl group through Dunnett’s test.

Based on an integrated bioinformatics approach using data acquired from three major databases, we explored the downstream target of RORα. Initially, we screened all potential transcriptional binding targets of RORα from the ChIP Atlas database (https://chip-atlas.org/), yielding a comprehensive list of genes directly regulated by RORα. We further investigated these candidates using the KEGG pathway database (https://www.kegg.jp/kegg/) and specifically selected genes involved in the NF-κB signaling pathway to focus on targets with potential relevance to inflammatory responses. Finally, we further refined our candidate list by screening for the main macrophage membrane proteins through the UniProt database (https://www.uniprot.org/). We identified TLR4 as a key downstream target of RORα through systematic integration of these three independent datasets (Figure 2A).

We first investigated the effect of hericenone C on *Tlr4* expression using RAW264.7 cells treated with hericenone C; *Tlr4* expression was significantly downregulated after 1 or 2 h of treatment (Figures 2B,C). We determined whether *Tlr4* downregulation was mediated through RORα using both pharmacological and genetic approaches. Co-treatment with hericenone C and the synthetic RORα-antagonist SR3335 synergistically downregulated *Tlr4* expression (Figure 2E). Furthermore, in RORα-overexpressing cells, hericenone C effectively counteracted the RORα-induced upregulation of *Tlr4*. Conversely, in RORα-knockdown cells, the suppressive effect of hericenone C on *Tlr4* expression was still evident (Figures 2D,F). These results collectively indicate that hericenone C modulates *Tlr4* expression primarily by targeting RORα.

**FIGURE 3:**
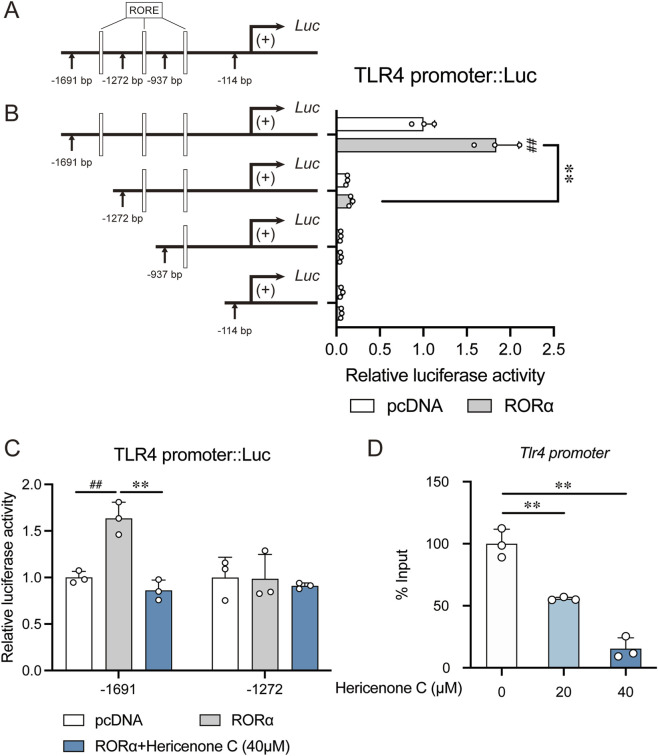
Effect of hericenone C on RORα binding to *Tlr4* promoter. **(A)** Schematic diagram showing the truncated *Tlr4* promoter::Luc constructs and the predicted RORE sites. **(B)** Luciferase activity of different *Tlr4* promoter constructs upon RORα overexpression in NIH3T3 cells. **(C)** Luciferase activity of the *Tlr4* promoter::Luc constructs (from −1,691 or −1,272 bp to +154 bp) in NIH3T3 cells co-transfected with RORα and treated with 40 µM of hericenone C. **(D)** Effect of hericenone C on binding affinity between RORα and *Tlr4* promoter. In all panels, each value represents the mean ± SD derived from three independent experiments (*n* = 3). **(B,C)** ^##^ *p* < 0.01 compared the RORα-overexpression group with the pcDNA group through an unpaired Student’s t-test. **p < 0.01 compared to the RORα-overexpression group through Dunnett’s test. **(D)** **p < 0.01 compared to 0 µM of the hericenone C group through Dunnett’s test.

Next, we investigated the precise molecular mechanism underlying the RORα-mediated regulation of *Tlr4* transcription. Bioinformatic analysis predicted potential ROREs within the *Tlr4* promoter region (Figure 3A). A series of luciferase reporter assays using truncated *Tlr4* promoter constructs revealed the crucial role of the fragment between −1,691 and −1,272 in RORα-driven transcriptional activation; the deletion of this segment abolished the response (Figure 3B). Importantly, hericenone C treatment significantly suppressed the RORα-enhanced luciferase activity regulated by the full-length promoter but did not influence the truncated promoter lacking the key responsive region (Figure 3C). Finally, ChIP analysis confirmed that hericenone C treatment markedly reduced RORα enrichment specifically on this *Tlr4* promoter fragment spanning from −1,691 to −1,272 (Figure 3D).

In summary, these data demonstrate that hericenone C specifically targets RORα to inhibit *Tlr4* transcription by inhibiting the binding of RORα to a specific response element within the *Tlr4* promoter.

### 3.3 Formalin activates the NF-κB signaling pathway through TLR4

**FIGURE 4:**
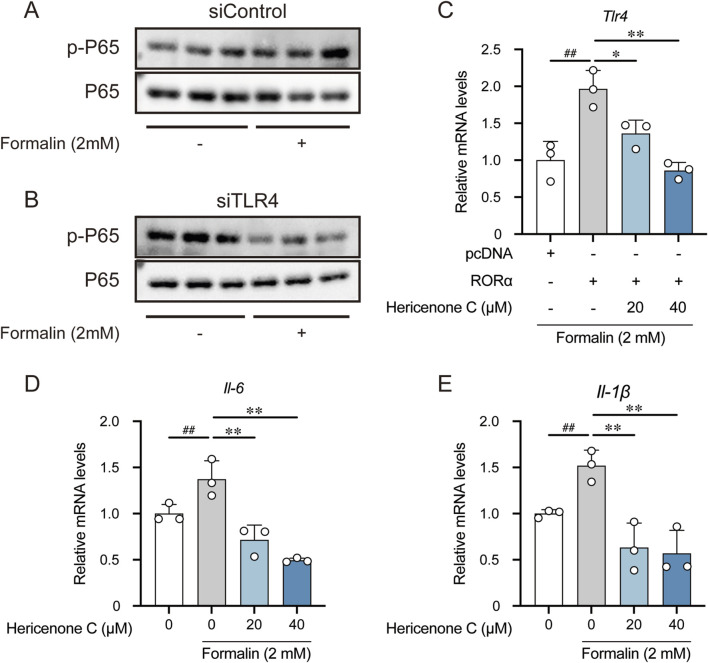
Effect of formalin on NF-κB pathway activation. **(A,B)** Effect of formalin on P65 phosphorylation in siControl **(A)** and siTLR4 **(B)** groups. **(C)** Regulation of hericenone C on *Tlr4* mRNA expression in formalin-treated RORα-overexpressing RAW264.7 cells. **(D,E)** Effect of hericenone C and formalin on the mRNA expression levels of **(D)** *Il-6* and **(E)** *Il-1β*. In C-E panels, each value represents the mean ± SD derived from three independent experiments (n = 3). **(C)** ^##^ *p* < 0.01 compared RORα-overexpression with pcDNA groups by unpaired Student’s t-test. **p < 0.01, *p < 0.05 indicate significant differences compared to the RORα-overexpression group (Dunnett’s test). **(D,E)** ^##^ *p* < 0.01 compared the formalin-treated group with the control group through unpaired Student’s t-test. **p < 0.01 compared to the formalin-treated group through Dunnett’s test.

To investigate whether formalin-induced inflammatory responses involve TLR4-mediated NF-κB activation, we transfected RAW264.7 cells with either siControl or siTLR4. Western blotting analysis revealed that formalin treatment slightly increased the ratio of phosphorylated P65 (p-P65) to total P65 in the siControl group, whereas this effect was markedly attenuated in the siTLR4 group (Figures 4A,B). Therefore, formalin promotes NF-κB pathway activation primarily through TLR4.

Considering that hericenone C suppressed TLR4 through RORα, we examined whether this mechanism interfered with formalin-induced inflammatory responses. In RORα-overexpressing RAW264.7 cells, formalin treatment significantly upregulated *Tlr4* expression, whereas hericenone C treatment effectively reversed this effect (Figure 4C). We further assessed the expression of key NF-κB downstream pro-inflammatory cytokines. Formalin treatment significantly upregulated the mRNA levels of *Il-6* and *Il-1β*, and hericenone C pre-treatment antagonized this effect in a dose-dependent manner (Figures 4D,E).

### 3.4 Macrophage depletion reduces formalin-induced second-phase nociception

**FIGURE 5:**
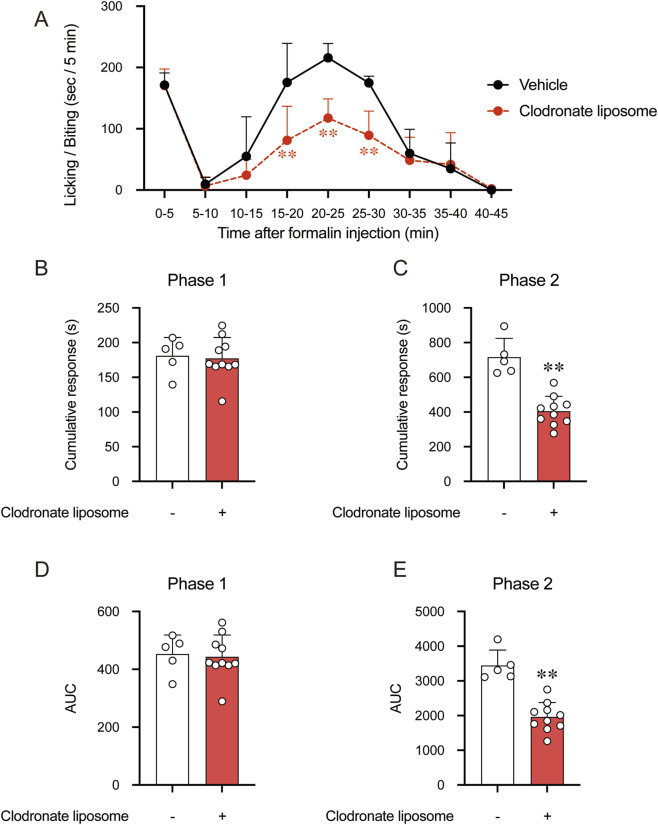
Effect of macrophage depletion on formalin-induced nociceptive behaviors in mice. **(A)** Time course of licking/biting time in seconds per 5 min after formalin injection. Clodronate liposomes were administered intraperitoneally 24 h before formalin injection. **(B,C)** Total licking/biting time in **(B)** phase 1 (0–10 min) and **(C)** phase 2 (10–45 min). **(D,E)** Calculating the area under the licking/biting time-time curve in **(D)** phase 1 and **(E)** phase 2. In **(B–E)**, each value represents an individual mouse (*n* = 5 or 10). **p < 0.01 compared the 25 mg/kg clodronate liposomes group with the vehicle group at the corresponding time points *via* one-way ANOVA with Tukey–Kramer *post hoc* tests. **p < 0.01 compared the 25 mg/kg clodronate liposomes group with the vehicle group *via* unpaired Student’s t-test.

To investigate the involvement of macrophages in the formalin-induced pain model, we depleted macrophages in mice using clodronate liposomes (*i.p.*), with vehicle liposomes (*i.p.*) serving as the control. After 24 h of treatment, formalin was injected subcutaneously into the hind paw, and pain behavior was recorded. The results revealed no significant effect of macrophage depletion on phase 1 of formalin-induced pain but markedly suppressed phase 2 (Figure 5A). This finding aligns with our previous report (Li et al., 2024), reflecting that hericenone C inhibits the recruitment of CD11c-positive cells at the formalin injection site. These results further validate the critical role of macrophages in mediating the sustained inflammatory pain response in the formalin model.

### 3.5 Hericenone C suppresses TLR4 expression in CD11c^+^ myeloid cells at formalin-injected paw tissues

**FIGURE 6:**
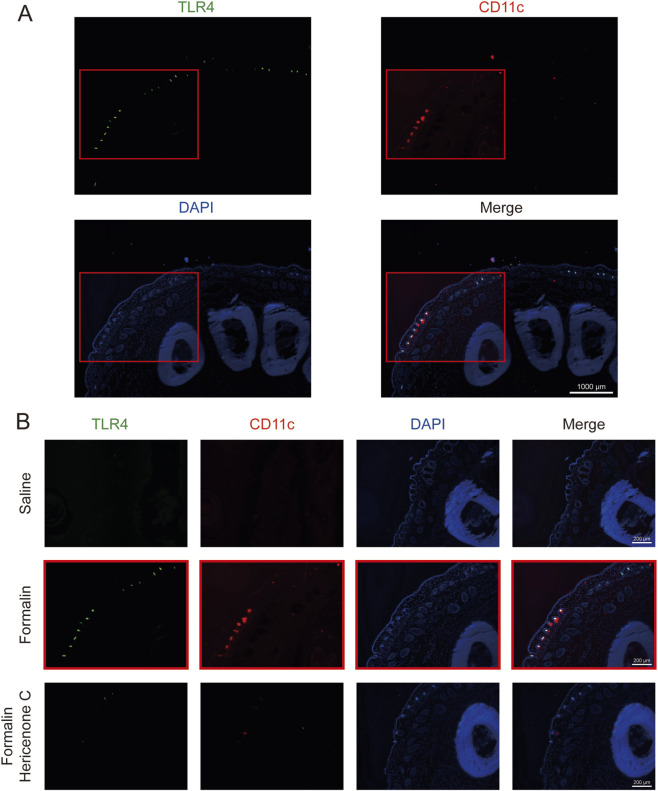
Effects of hericenone C on TLR4 expression in formalin-injected paw tissues. **(A,B)** Representative images of the fluorescent immunostaining assay performed to identify TLR4-expressing cells obtained from mouse paw coronal sections. All images have the same settings for TLR4 (green), CD11c (red), and DAPI (blue). Scale bars are indicated on each image. **(A)** Representative whole image of a paw obtained from formalin-treated mice. The parts boxed in red were trimmed and used in the formalin group in **(B)**.

Based on our *in vitro* findings, we hypothesized that hericenone C might inhibit TLR4 expression during formalin-induced *in vivo* inflammation. The immunofluorescence co-staining of CD11c (a myeloid cell marker) and TLR4 conducted to confirm this hypothesis revealed that TLR4 expression was localized to CD11c^+^ cells at the peripheral regions of inflamed tissues after formalin treatment (Figure 6A). Pretreatment with hericenone C reduced CD11c^+^ cell infiltration as well as significantly decreased TLR4 levels in these cells (Figure 6B). These results demonstrate that hericenone C systemically inhibits TLR4-mediated inflammatory responses by targeting CD11c^+^ cell recruitment and TLR4 expression *in situ*.

### 3.6 RORα promotes formalin-induced nociception in monocyte-enriched PBMCs

To investigate whether RORα activity within monocytes modulates nociception in the local tissue environment, we used an adoptive transfer model using PBMCs. For this experiment, the isolated monocyte-enriched PBMCs were injected into the subcutaneous tissue of the hind paw 5 min before formalin administration.

**FIGURE 7:**
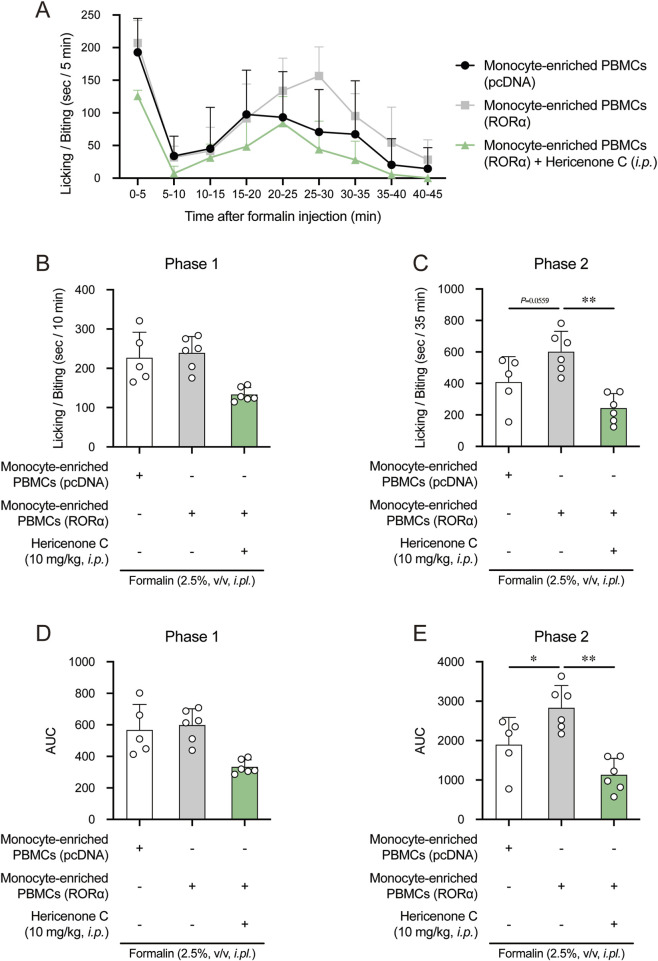
Effect of hericenone C and RORα-overexpressing monocyte-enriched PBMCs on phase 2 nociception. **(A)** Time course of licking/biting time in seconds per 5 min after formalin injection. Monocyte-enriched PBMCs were administered to the different sites in the same formalin-injected paw 5 min before formalin injection. **(B,C)** Total licking/biting time of **(B)** phase 1 (0–10 min) and **(C)** phase 2 (10–45 min). **(D,E)** Calculating the area under the licking/biting time-time curve in **(D)** phase 1 and **(E)** phase 2. In B-E panels, each value represents an individual mouse (*n* = 5 or 6). **p < 0.01 compared RORα-overexpressing monocyte-enriched PBMCs group with the pcDNA-transfected monocyte-enriched PBMCs group at the corresponding time points by one-way ANOVA with Tukey–Kramer *post hoc* tests. **p < 0.01 compared the RORα-overexpressing monocyte-enriched PBMCs group with the pcDNA-transfected monocyte-enriched PBMCs group through unpaired Student’s t-test.

**FIGURE 8:**
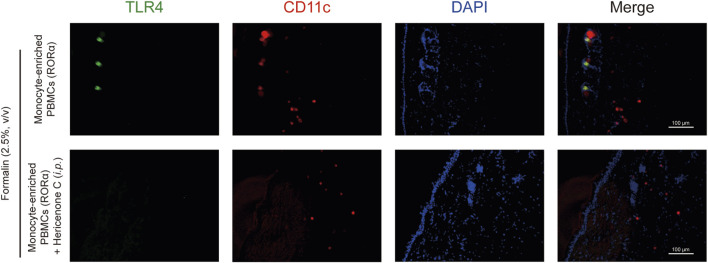
Effects of hericenone C on TLR4 expression in RORα-overexpressing PBMCs and formalin-injected paw tissues. Representative images of fluorescent immunostaining performed to identify TLR4-expressing cells obtained from mouse paw coronal sections. All images have the same settings for TLR4 (green), CD11c (red), and DAPI (blue). Scale bars are indicated on each image. Representative whole image of a paw obtained from formalin-treated mice.

First, RORα was overexpressed in monocyte-enriched PBMCs through transfection. Mice receiving RORα-overexpressing PBMCs exhibited significantly exacerbated second-phase nociceptive behavior compared to their control (pcDNA-transfected PBMCs) counterparts. Systemic pretreatment with hericenone C (10 mg/kg) reversed this pro-nociceptive effect (Figures 7B,D). Immunofluorescence analysis of the injected paw tissues revealed that RORα overexpression induced a strong TLR4 signal on CD11c^+^ cells, which was abolished by hericenone C treatment (Figure 8). The efficacy of RORα overexpression in upregulating *Tlr4* in the donor PBMCs was confirmed by qPCR (Supplementary Figure S10C).

**FIGURE 9:**
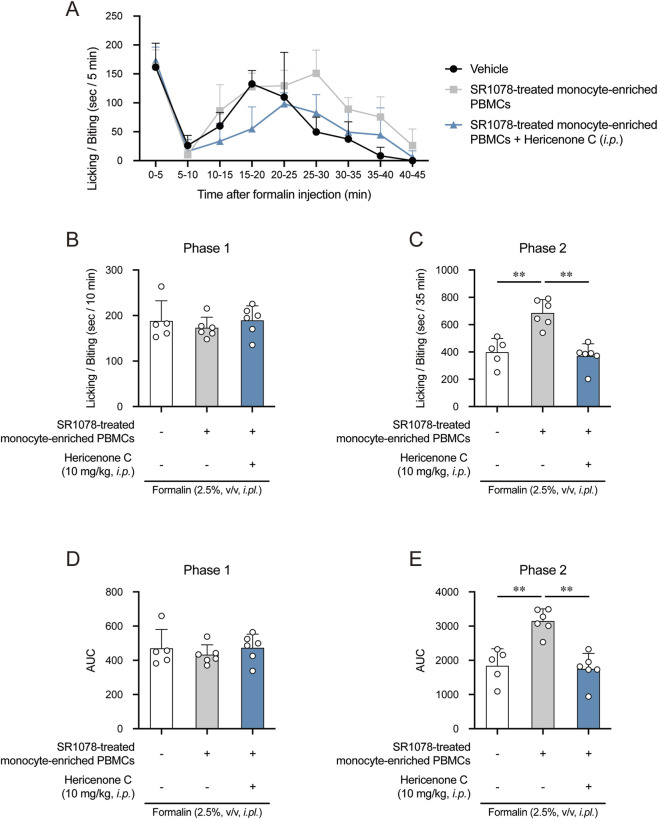
Effect of hericenone C and SR1078-treated monocyte-enriched PBMCs on phase 2 nociception. **(A)** Time course of licking/biting time in seconds per 5 min after formalin injection. Monocyte-enriched PBMCs were administered to the different sites in the same formalin-injected paw 5 min before formalin injection. **(B,C)** Total licking/biting time in **(B)** phase 1 (0–10 min) and **(C)** phase 2 (10–45 min). **(D,E)** Calculating the area under the licking/biting time-time curve in **(D)** phase 1 and **(E)** phase 2. In B-E panels, each value represents an individual mouse (*n* = 5 or 6). **p < 0.01 compared the SR1078-treated monocyte-enriched PBMCs group with the vehicle group at the corresponding time points through one-way ANOVA with Tukey–Kramer *post hoc* tests. **p < 0.01 compared the SR1078-treated monocyte-enriched PBMCs group with the vehicle group through unpaired Student’s t-test.

To further corroborate this finding, we pre-activated RORα in donor PBMCs using the agonist SR1078. Mice receiving SR1078-treated PBMCs also showed enhanced second-phase pain, which was suppressed by hericenone C pretreatment, as described above (Figures 9B,D). Thus, the upregulation of *Tlr4* in these SR1078-treated PBMCs was verified (Supplementary Figure S10A). The pain behavior did not significantly differ between the control PBMC groups analyzed in both experiments (Supplementary Figure S11).

Therefore, increased RORα activity in monocytes sufficiently exacerbates formalin-induced nociception, and hericenone C can mitigate this effect. The data support a model whereby hericenone C mediates antinociception, at least in part, through functional antagonism of RORα in monocytes.

## 4 Discussion

Previously, we demonstrated that hericenone C alleviates formalin-induced second-phase nociception; the present study characterizes the direct binding target of hericenone C with RORα. RORα, RORβ, and RORγ belong to the retinoic-acid-related orphan receptor (ROR) family, forming a subgroup of nuclear receptors (Nematisouldaragh et al., 2024a; Wang et al., 2024). The transcription factor RORα binds to ROR response elements (ROREs) in the promoter regions of target genes, functioning in a monomeric manner (Vitalis and Mariani, 2018). A previous report has indicated variable functions of RORα across different immune cells. In inflammatory bowel disease, RORα promotes T cell survival and mTORC1 activation, leading to the infiltration of T-cells in the lesion site, and induces gut inflammation (Chi et al., 2021). RORα deletion in group 2 innate lymphoid cells (ILC2s) affects the abundance of gastrointestinal ILC2s and Group 2 innate lymphoid cells (ILC3s), and enhances T helper 17 (Th17)-type responses, whereas RORα deletion in ILC3/Th17 cells suppresses IL-17 production, mitigating intestinal fibrosis and inflammation (Kabil et al., 2025). Moreover, RORα directly binds to STAT3 and transcriptionally upregulates *Il6* through RORE, resulting in STAT3 phosphorylation and exacerbating cartilage damage (Liang et al., 2021). The pro-inflammatory functions of RORα can be modulated by synthetic ligands, as demonstrated by the inverse agonist SR3335 that suppresses the expression of pro-inflammatory cytokines, such as IL-17 (Nematisouldaragh et al., 2024b). In this study, we also validated the functions of existing RORα ligands. SR1078 upregulated *Tlr4* expression in monocyte-enriched PBMCs (Supplementary Figure S10A), whereas SR3335 downregulated it in RAW264.7 cells (Figure 2E). Furthermore, these findings revealed the immunomodulatory function of RORα in macrophages, which facilitates the inflammatory pain by regulating the TLR4 expression. This is the first study that identifies a novel natural compound serving as a selective RORα antagonist, highlighting its potential as a lead compound for drug development and a unique tool for probing RORα biology.

RORα does not directly participate in NF-κB signaling; however, through the integrated bioinformatic analysis, we identified TLR4 as the crucial molecular bridge, a macrophage membrane receptor that is transcriptionally regulated by RORα and functionally integrated into NF-κB pathways. TLR4, a well-characterized mediator of inflammatory pain, is activated by damage-associated molecular patterns (DAMPs) and drives NF-κB-dependent cytokine production *via* P65 phosphorylation (Kim et al., 2023; Rayees et al., 2020; Zhang et al., 2022). Previous studies have implicated the analgesic effect of the TLR4 antagonist in formalin-induced pain. PGA, a natural antagonist of TLR4, which originates from *Rhizobium leguminosarum* and structurally resembles LPS lipid A, inhibits both LPS-induced NF-κB activation and formalin-induced phase 2 nociception (Iannotta et al., 2021); moreover, cannabidivarin from *Cannabis sativa* attenuates pain behaviors and morphine tolerance through TLR4 antagonism in phase 2 (Wang et al., 2022). We detected hericenone C-mediated suppression of *Tlr4* transcription *via* reduced RORα promoter binding. Although our ChIP-qPCR data demonstrated a significant reduction in RORα occupancy at the *Tlr4* promoter upon hericenone C treatment, the concomitant decrease in *Tlr4* mRNA expression was more modest. A reduction in nascent transcription due to impaired RORα binding may require time to be fully reflected in the steady-state mRNA pool due to the kinetics of transcriptional regulation and mRNA turnover, and may also be partially compensated by contributions from other transcription factors regulating *Tlr4*. The rapid recovery of *Tlr4* expression upon hericenone C withdrawal and the establishment of a new steady-state mRNA level within 12 h of treatment reflected the transient and reversible nature of this transcriptional suppression. Notably, we confirmed TLR4 as the functional target of formalin, as TLR4 knockdown attenuated formalin-induced P65 phosphorylation, while hericenone C reversed formalin-driven TLR4 upregulation and CD11c^+^ cell recruitment. These findings position the RORα-TLR4-NF-κB axis as a druggable cascade for inflammatory pain.

Our data confirm the stage-specific role of macrophages in formalin-induced inflammatory pain. While phase 1 nociception is primarily neurogenic, the efficacy of both macrophage depletion and hericenone C in selectively suppressing phase 2 highlights the centrality of CD11c^+^ cells in sustained inflammatory sensitization. Critically, we demonstrate that RORα activity in these cells majorly drives this process. This correlation was validated through the adoptive transfer model, reflecting monocyte-enriched PBMCs, either overexpressing RORα or pretreated with SR1078, significantly exacerbated phase 2 nociception. The hericenone C-mediated complete reversal of this nociceptive effect identifies it as a functional antagonist of this pathway *in vivo*. This mechanistic insight is further refined at the site of inflammation, where the infiltrating CD11c^+^ cells exhibited high TLR4 expression (Figure 6A). The dual action of hericenone C by reducing both CD11c^+^ cell recruitment and intra-tissue TLR4 levels (Figure 6B) suggests it disrupts a self-reinforcing, RORα/TLR4-driven positive feedback loop that amplifies and sustains the inflammatory pain signal.

The mechanistic variations of hericenone C from conventional analgesics underpin its potential therapeutic value. Non-steroidal anti-inflammatory drugs (NSAIDs) non-selectively inhibit cyclooxygenase (COX) enzymes, disrupting prostaglandin synthesis broadly and posing risks of gastrointestinal and cardiovascular side effects (Bindu et al., 2020). Opioids, acting primarily on central nervous system μ-opioid receptors, effectively blunt pain perception but carry high risks of addiction, tolerance, and respiratory depression (Abdel Shaheed et al., 2024). Contrastingly, hericenone C targets the RORα-TLR4 axis in macrophages, suggesting a potentially favorable side effect profile by avoiding widespread inhibition of prostaglandins or central opioid receptors. Furthermore, it offers a targeted strategy for managing inflammatory pain, potentially filling a gap between non-specific NSAIDs and centrally addictive opioids.

Given the conserved core structure among hericenones, we hypothesized that their bioactivity resides primarily in the core scaffold rather than the variable fatty acid side chain. To test this while generating a probe for pull-down assays, we conjugated biotin specifically to the side chain of hericenone C (Supplementary Figure S3). Importantly, the biotinylated hericenone C exhibited indistinguishable activity from the native compound through luciferase reporter assay in NRE::Luc expressing NIH3T3 cells, with no statistically significant difference in their inhibitory effects (Supplementary Figure S2). This result provides functional validation that our design strategy was successful and offers evidence that the fatty acid side chain is not involved in critical binding interactions with RORα. Therefore, we conclude that the core scaffold of hericenone C is responsible for engaging the RORα ligand-binding domain, a hypothesis to be confirmed by future structural studies.

A technical consideration in our *in vitro* studies is the challenge posed by the low basal expression of RORα in RAW264.7 cells. The generation of stable gain-of-function or loss-of-function cell models was limited by low transfection efficiency, a common issue associated with macrophage cell lines. Consequently, we relied on transient transfection approaches, which may lead to heterogeneous expression and variable downstream responses. This inherent technical limitation likely accounts for the comparatively moderate changes in some transcriptional and functional outcomes, despite the associated statistical and biological significance. Our study focuses on macrophages, whereas further analysis can determine whether hericenone C also modulates RORα in other immune cells (e.g., microglia or neutrophils), contributing to the chronicity of pain. Besides, while our competitive proteomic profiling suggested a binding preference for RORα, as RORβ and RORγ were not enriched among the candidates, a definitive functional specificity profile against all ROR isoforms deserves future systematic investigation. Furthermore, future studies using conditional RORα knockout mice would effectively help confirm the cell-specificity of its action *in vivo*. Additionally, investigating the potential synergy between hericenone C and low-dose opioids could explore its utility in managing severe pain while minimizing opioid-related side effects.

In conclusion, this study demonstrates that hericenone C alleviates formalin-induced inflammatory pain by specifically targeting the RORα-TLR4 axis in macrophages. The comprehensive mechanistic studies revealed that hericenone C functions as a novel RORα antagonist, suppressing *Tlr4* transcription. Importantly, TLR4 is potentially a direct target of formalin and plays a critical role in mediating the second phase of formalin-induced pain. The dual action of hericenone C to inhibit both macrophage recruitment and TLR4 expression validates its role as a promising lead compound for developing targeted analgesics with potentially fewer side effects compared to those associated with conventional anti-inflammatory drugs.

## Data Availability

The original contributions presented in the study are included in the article/Supplementary Material, further inquiries can be directed to the corresponding authors.
