# Supplementary material for: Hericenone C exhibits anti-nociceptive effects through RORα-mediated suppression of TLR4 transcription
Source: Front Pharmacol. 2026 Mar 18;17:1703176. doi: 10.3389/fphar.2026.1703176 (PMC13038899; doi:10.3389/fphar.2026.1703176)
Supplement: Supplementary file 4 [file DataSheet1.docx]

Supplementary Material

# **1. Supplementary Figures**


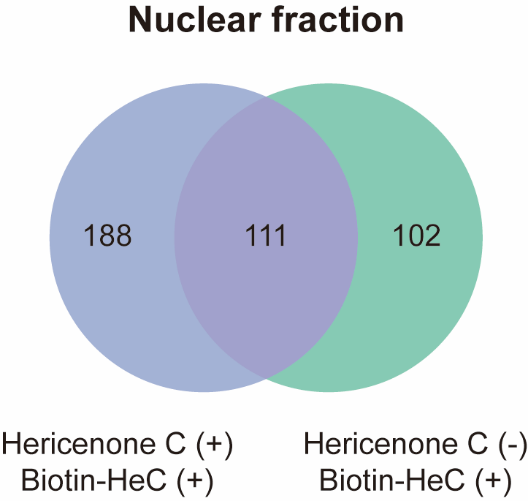


## **Supplementary Figure 1.** Venn diagram of the binding targets in the nuclear fraction. Hericenone C, a competitive inhibitor, was used in the 1-h pre-treatment to exclude unspecific binding targets.


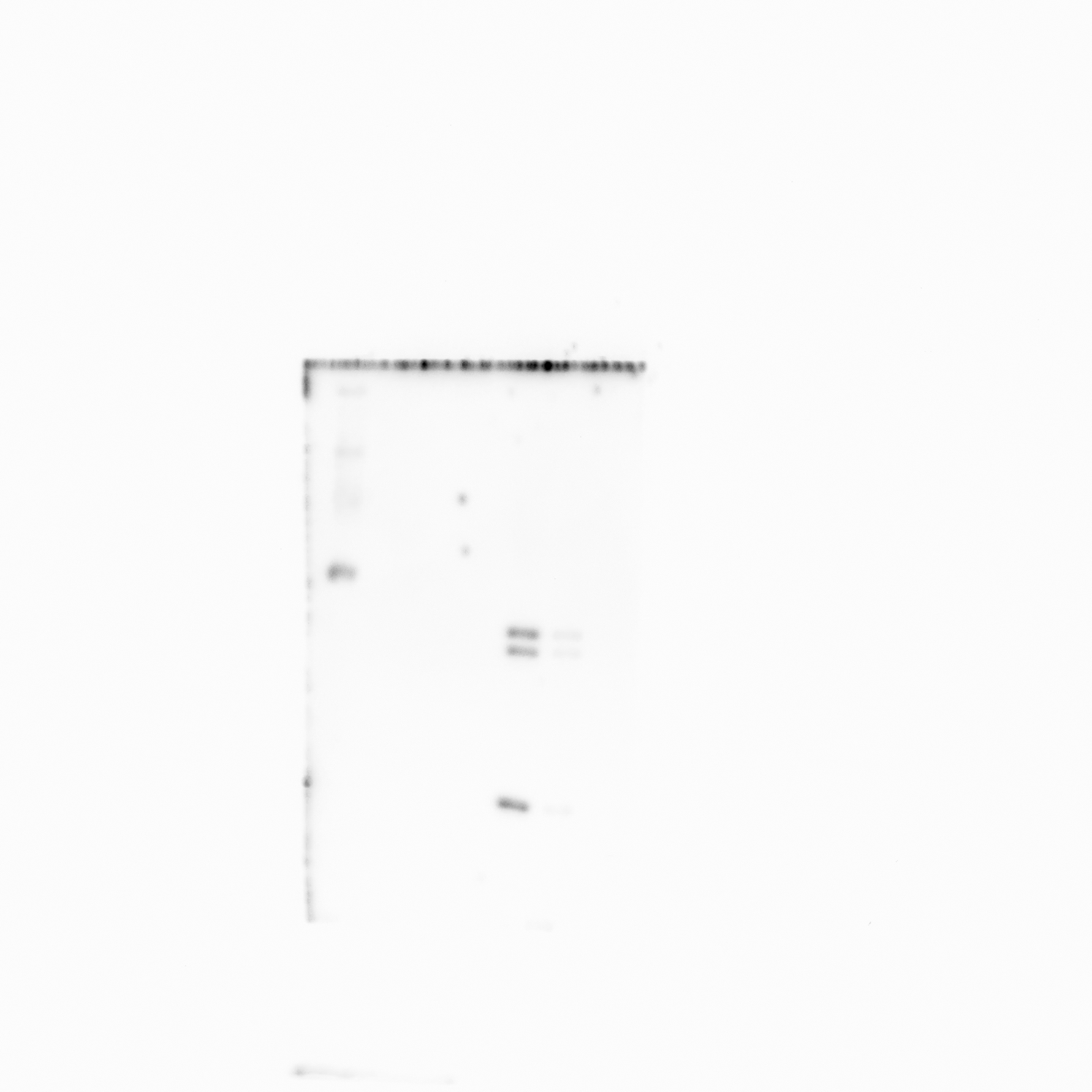


## **Supplementary Figure 2.** Original membrane related to Figure 1C.


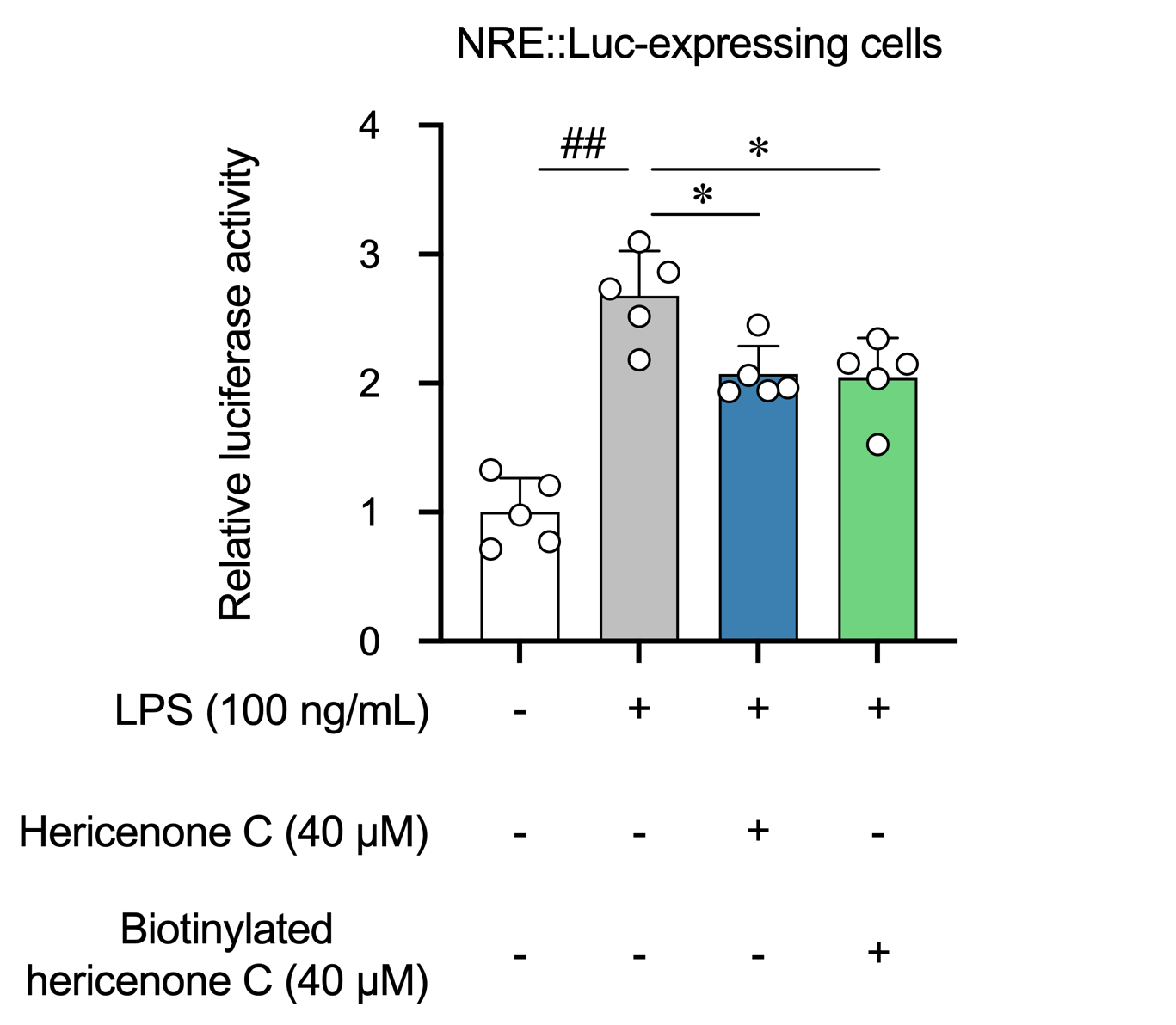


## **Supplementary Figure 3.** Effect of hericenone C and biotinylated hericenone C on luciferase activity in NF-κB response element (NRE)::Luciferase (Luc) expressing NIH3T3 cells induced by LPS (100 ng/mL).

*Each value represents the mean ± SD derived from five independent experiments (n = 5).*

*##p < 0.01 compared the LPS group with the control group through unpaired Student’s t-test. *p < 0.05 compared to the LPS group using Dunnett's test.*


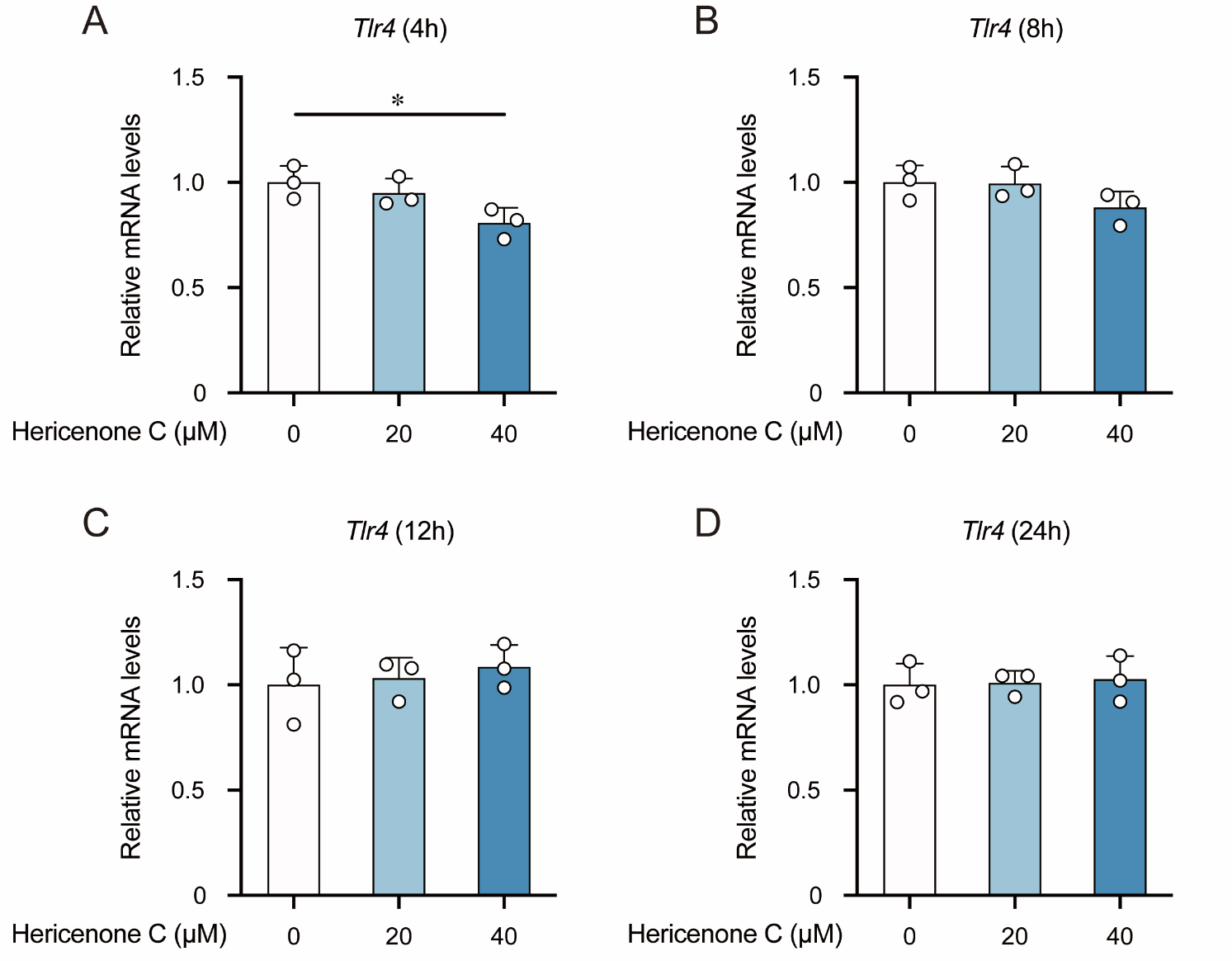


## **Supplementary Figure 4.** Effect of hericenone C on *Tlr4* mRNA expression for (A) 4 h, (B) 8 h, (C) 12 h, and (D) 24 h.

In all panels, each value represents the mean ± SD derived from three independent experiments (n = 3).

**p < 0.05 compared to 0 μM of the hericenone C group through Dunnett's test.*


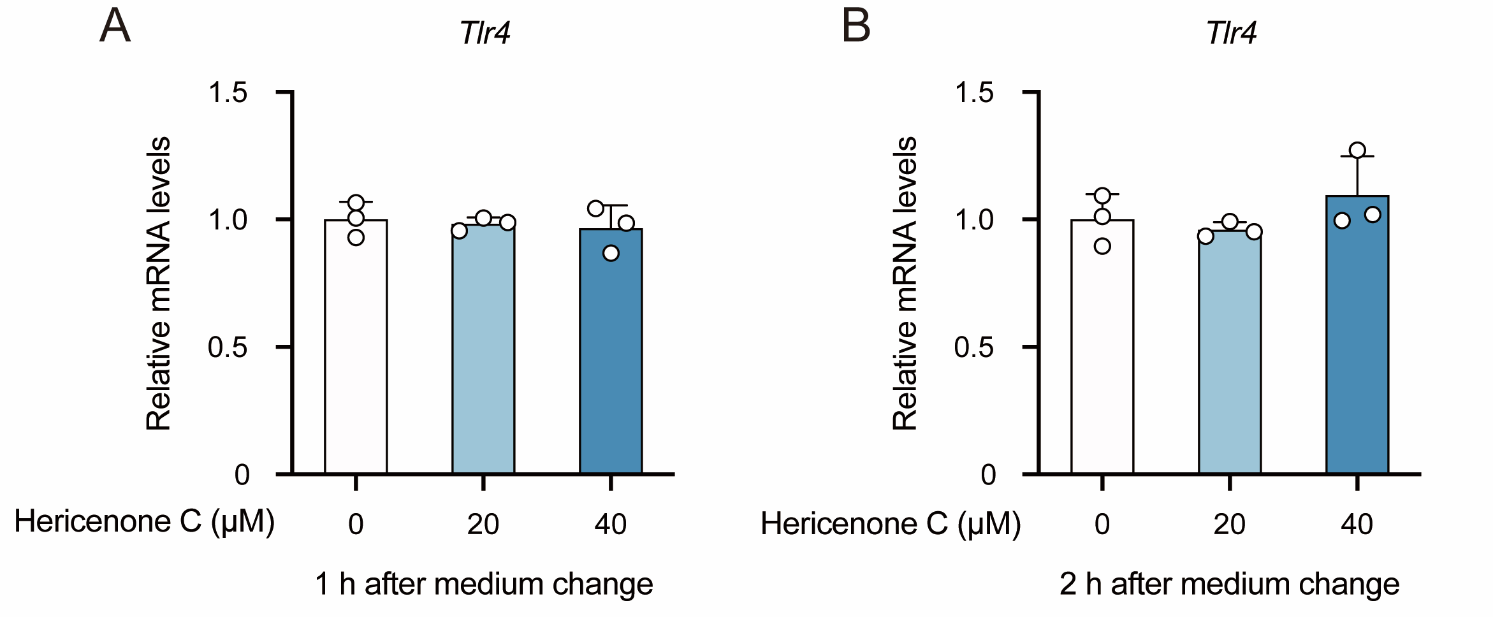


## **Supplementary Figure 5.** Effect of hericenone C removal for (A) 1 h and (B) 2 h on *Tlr4* mRNA expression. Hericenone C pretreatment (1 h) of RAW264.7 cells.

In all panels, each value represents the mean ± SD derived from three independent experiments (n = 3).


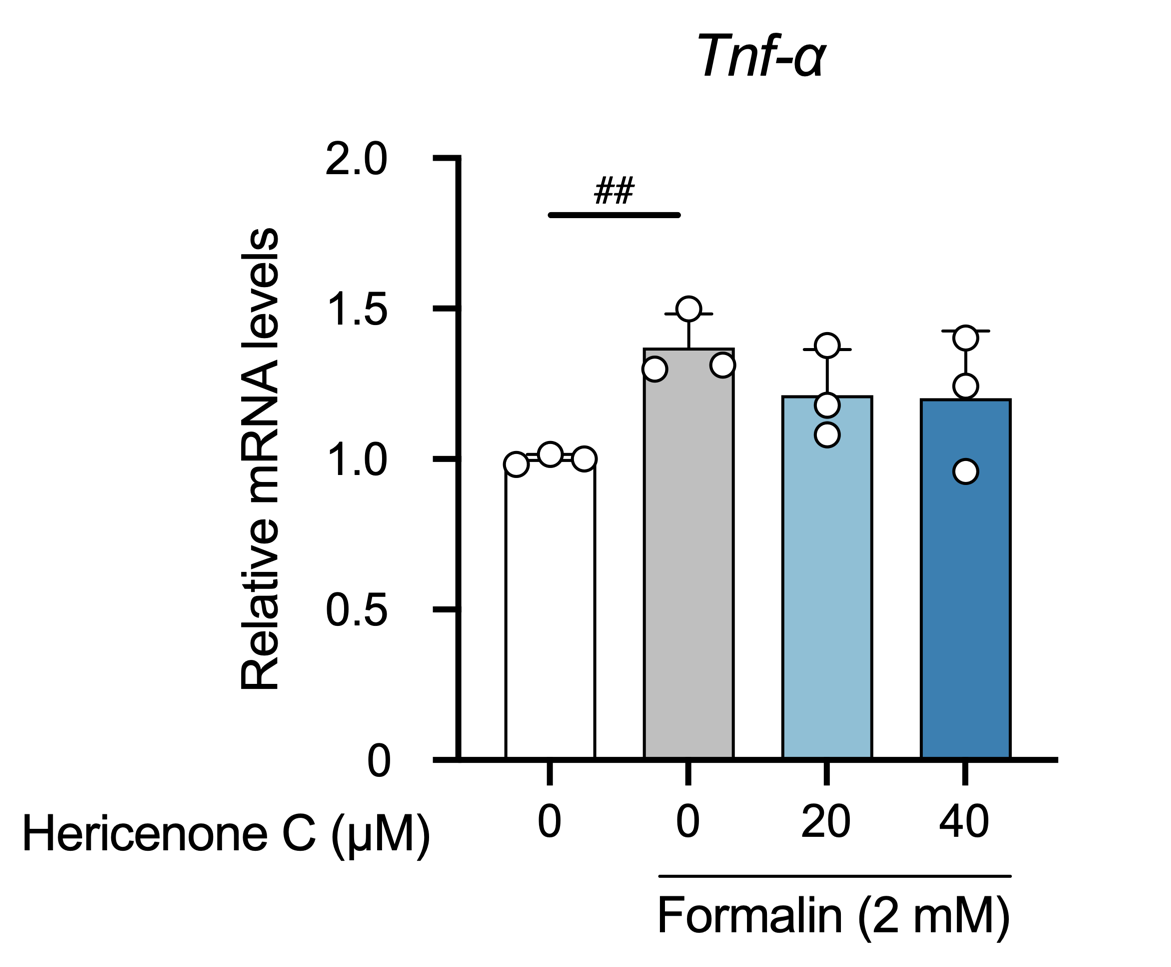


## **Supplementary Figure 6.** Effect of hericenone C and formalin on *Tnf-α* mRNA expression.

In all panels, each value represents the mean ± SD derived from three independent experiments (n = 3).


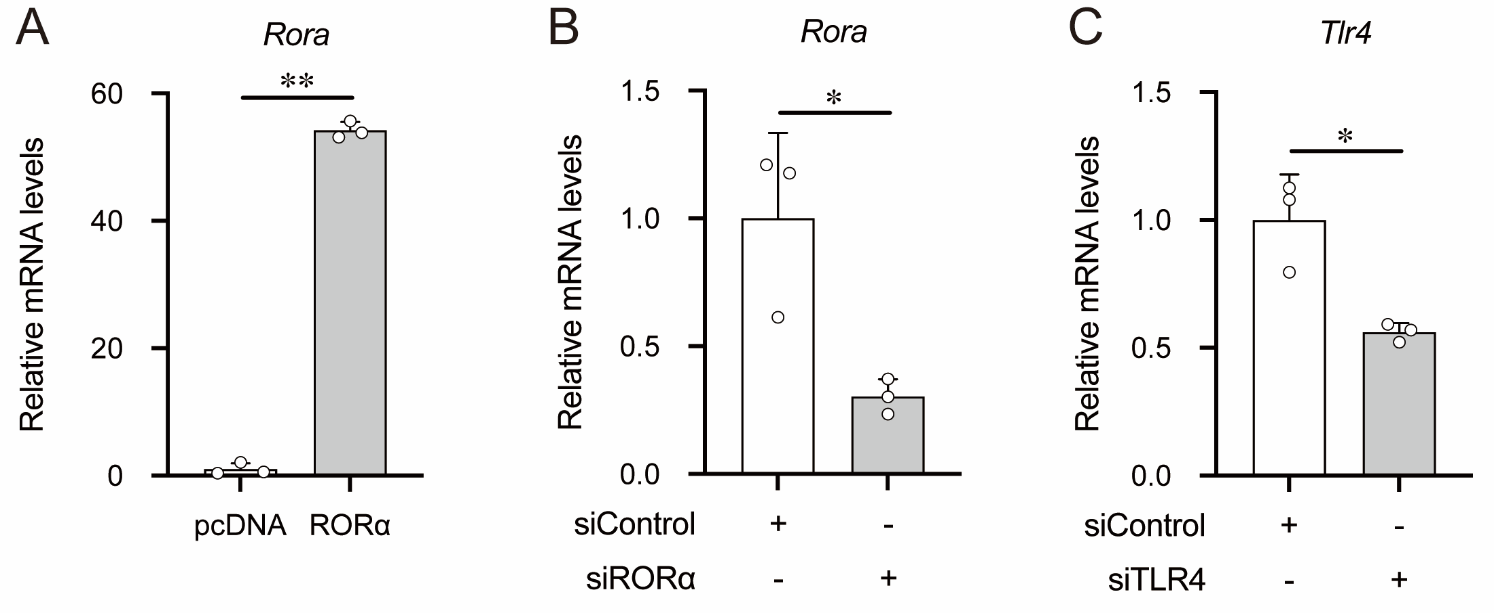


## **Supplementary Figure 7.** Validation of transfection efficiency for RORα and TLR4. qPCR analysis of (A) RORα overexpression, (B) RORα knockdown, and (C) TLR4 knockdown.

In all panels, each value represents the mean ± SD derived from three independent experiments (n = 3).

***p < 0.01, *p < 0.05 compared the two groups by Student’s t-test.*


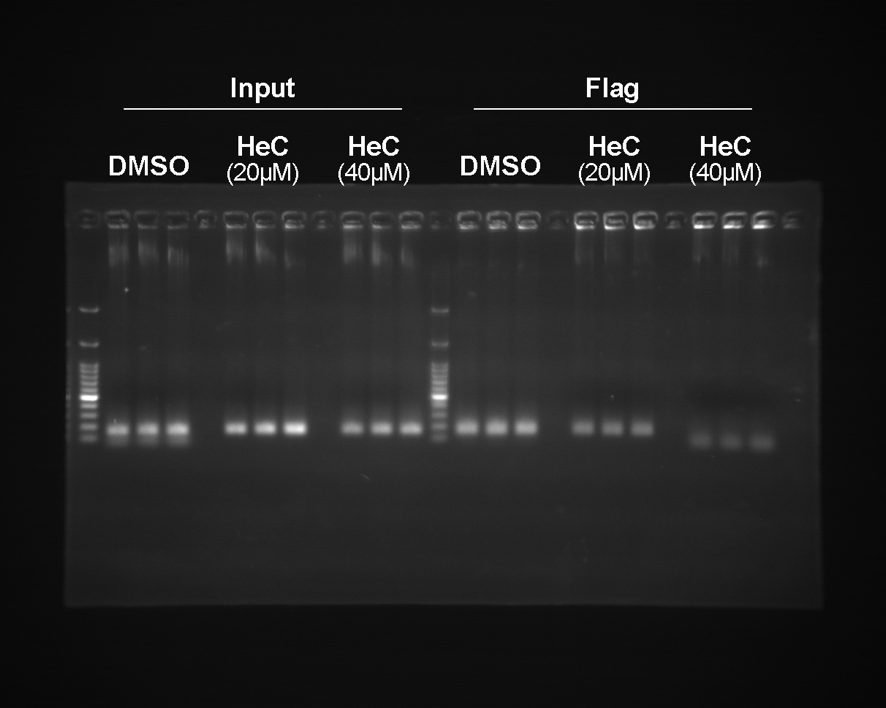


## **Supplementary Figure 8.** PCR electrophoresis gel related to Figure 2D.


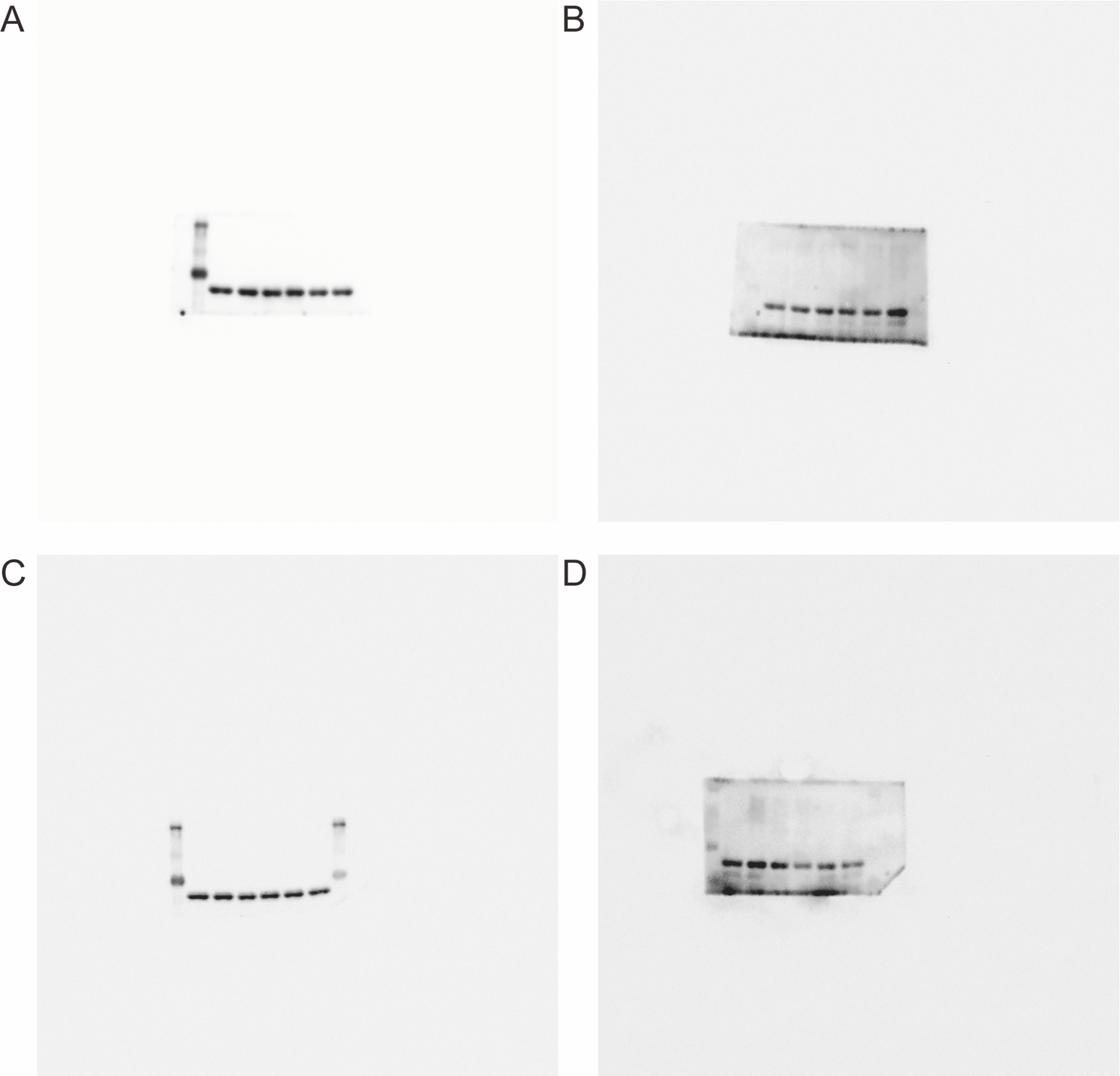


## **Supplementary Figure 9.** Original membrane of Figure 5A and 5B. (A, C) P65 and (B, D) p-P65.


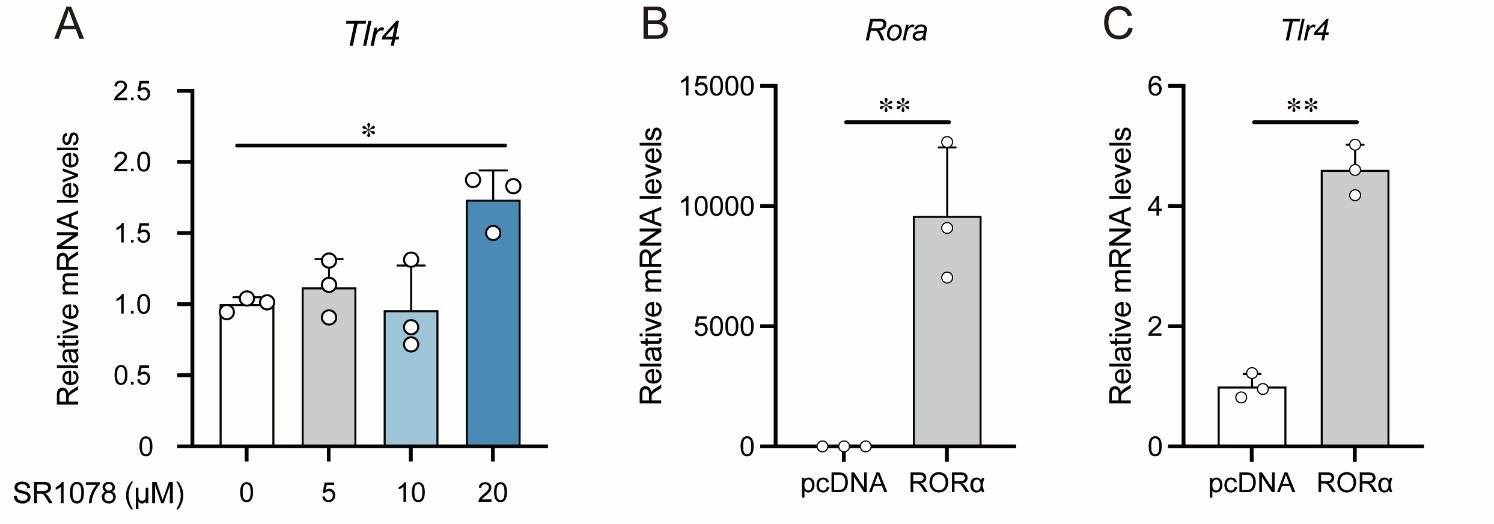


## **Supplementary Figure 10.** Effect of SR1078 or RORα overexpression on *Tlr4* mRNA expression in monocyte-enriched PBMCs. qPCR analysis of (A) *Tlr4* mRNA expression after SR1078 treatment (20 µM), (B) *Rora* mRNA expression, and (C) *Tlr4* mRNA expression by RORα overexpression.

In all panels, each value represents the mean ± SD derived from three independent experiments (n = 3).

***p < 0.01, *p < 0.05 compared the two groups by Student’s t-test.*


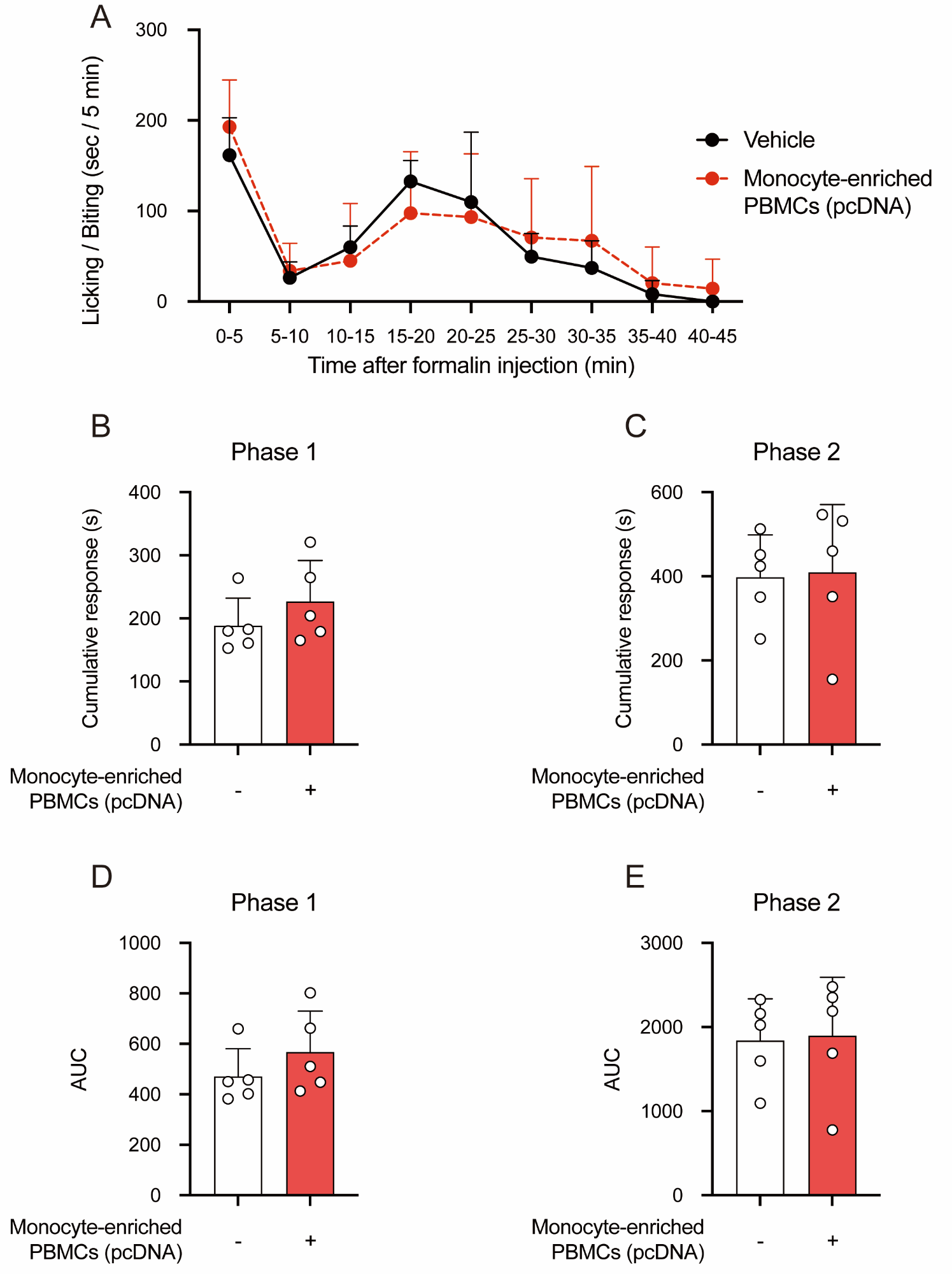


## **Supplementary Figure 11.** Effect of pcDNA-transfected monocyte-enriched PBMCs on formalin-induced nociceptive behaviors.

(A) Time course of licking/biting time in seconds per 5 min after formalin injection. Monocyte-enriched PBMCs were administered to the different sites in the same formalin-injected paw for 5 min before formalin injection.

(B, C) Total licking/biting time in (B) phase 1 (0–10 min.) and (C) phase 2 (10–45 min).

(D, E) Calculating the area under the licking/biting time-time curve in (D) phase 1 and (E) phase 2.

In B-E panels, each value represents an individual mouse (n = 5).


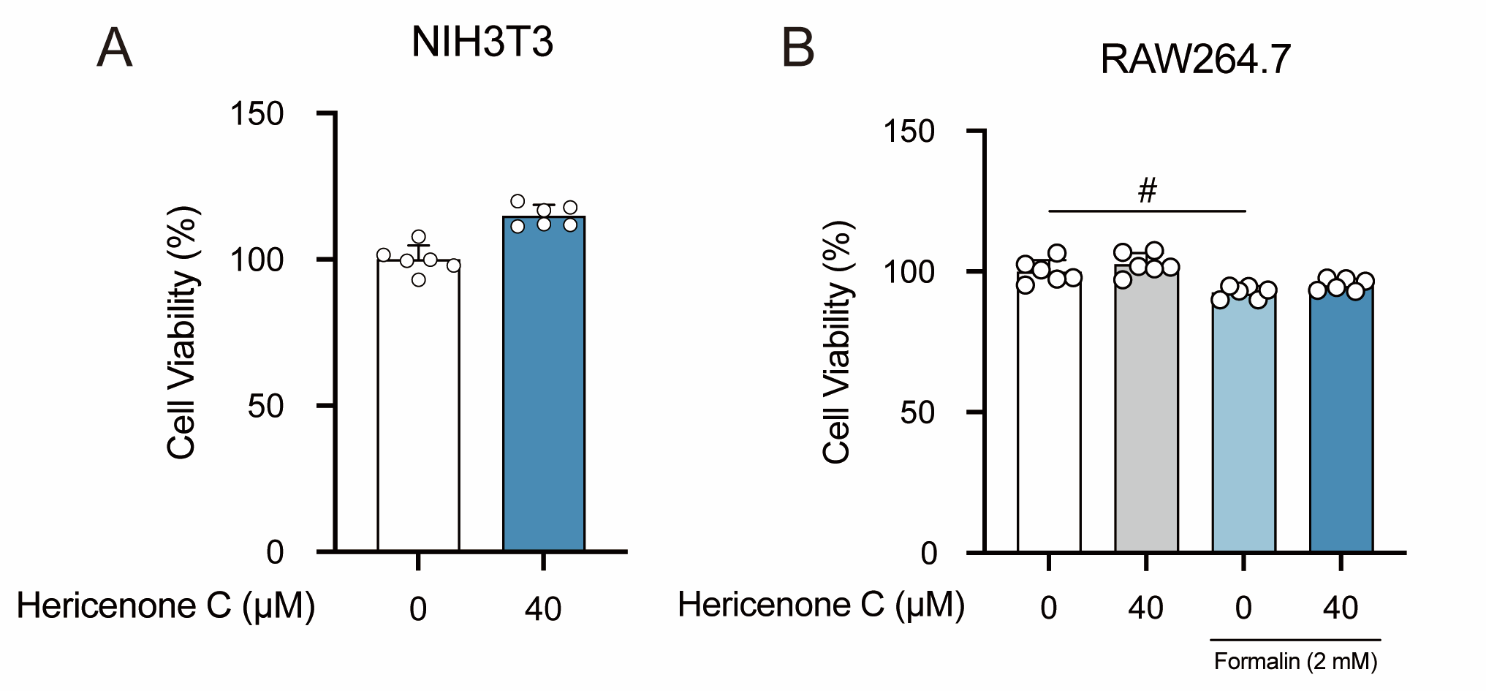


## **Supplementary Figure 12.** Effect of hericenone C and formalin on the viability of (A) NIH3T3 and (B) RAW264.7 cells.


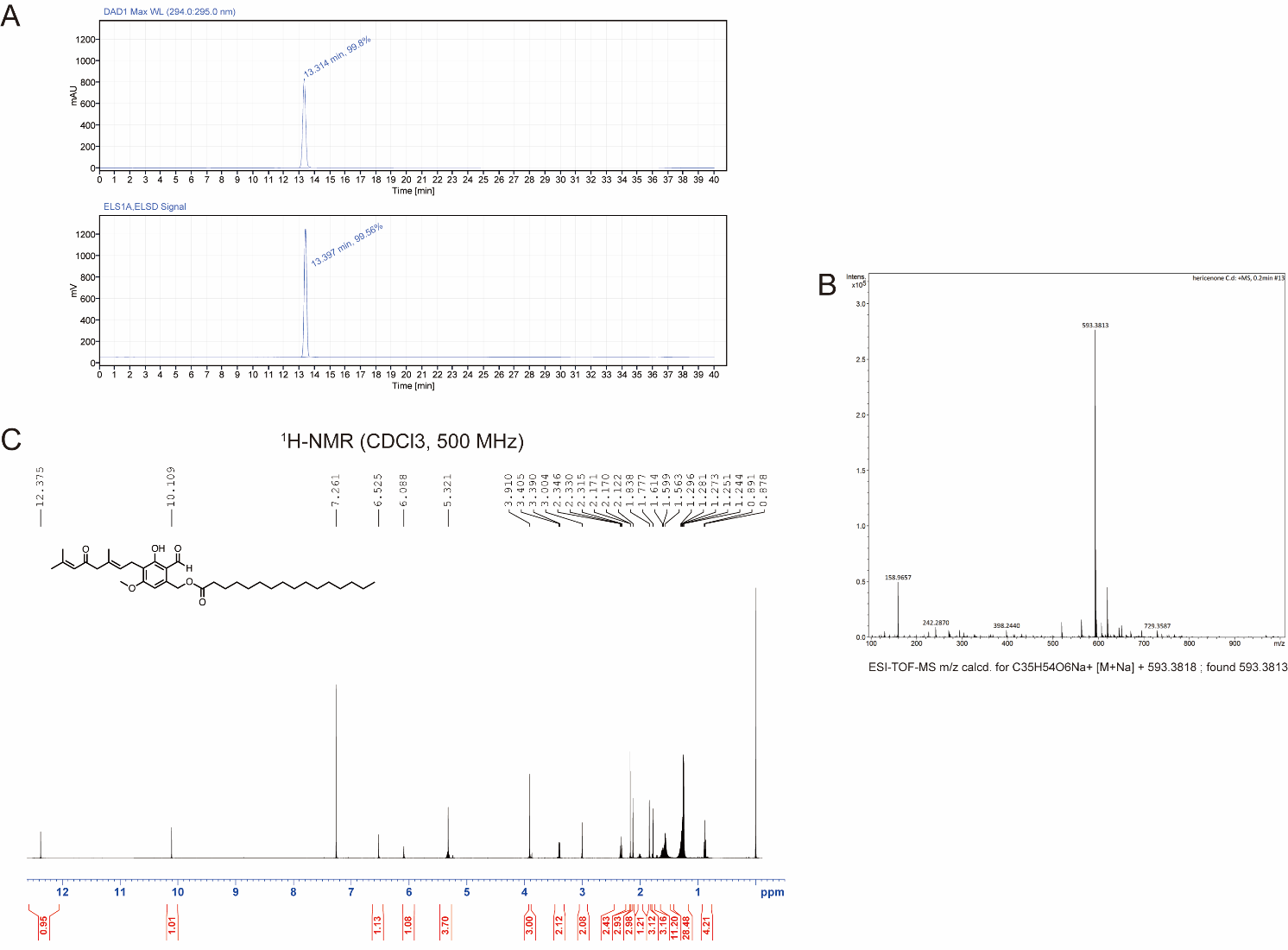


## **Supplementary Figure 13.** Identification of hericenone C. (A) Purity analysis by HPLC; identification via (B) MS and (C) NMR.


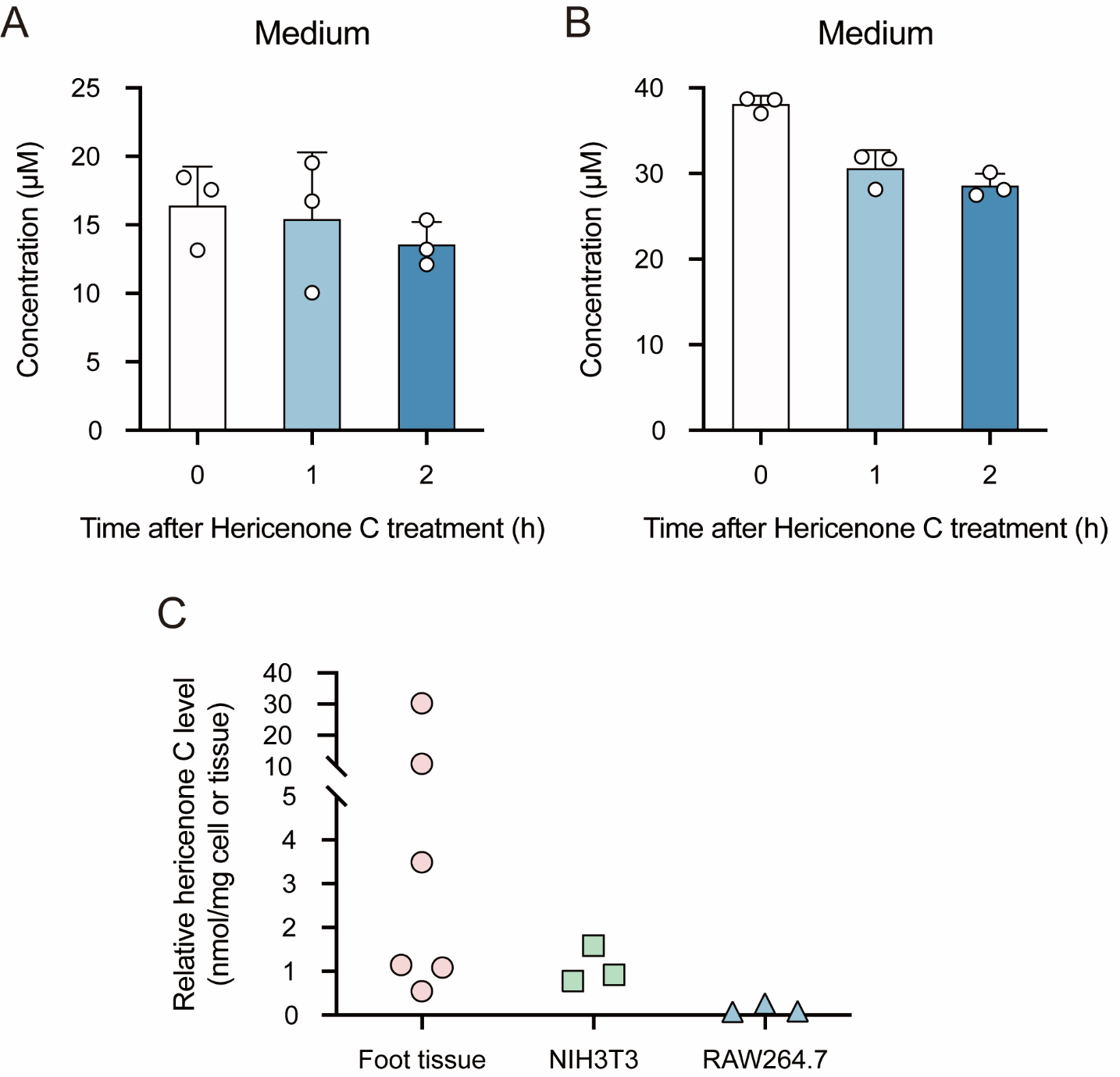


## **Supplementary Figure 14.** Stability of (A) 20 µM and (B) 40 µM of hericenone C in culture medium and (C) its distribution in cells and foot tissue.


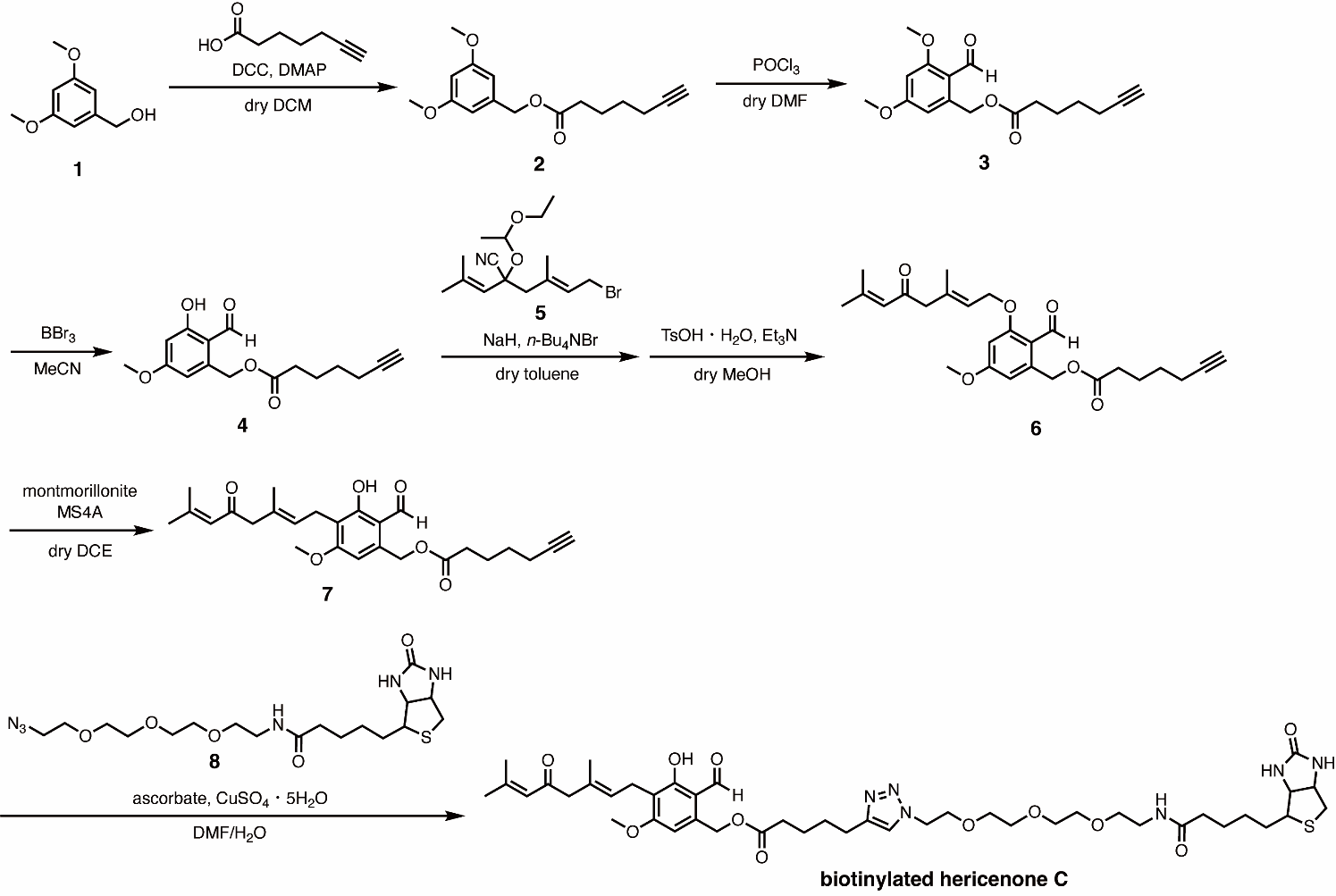


## **Supplementary Figure 15.** Synthesis of biotinylated hericenone C.

# **2. Supplementary Tables**

## **Supplementary Table 1**. Primer sets for Quantitative RT-PCR, cloning, and ChIP-PCR analysis.

## **Supplementary Table 2.** Binding targets of hericenone C in cytosolic and nuclear fractions.

## **Supplementary Table 3.** Gene lists from the bioinformatic database for RORα downstream target screening.
